# Genome-Wide Identification and Expression of *Xenopus* F-Box Family of Proteins

**DOI:** 10.1371/journal.pone.0136929

**Published:** 2015-09-01

**Authors:** Banu Saritas-Yildirim, Hannah A. Pliner, Angelica Ochoa, Elena M. Silva

**Affiliations:** Department of Biology, Georgetown University, Washington, DC, United States of America; University of Colorado, Boulder, UNITED STATES

## Abstract

Protein degradation via the multistep ubiquitin/26S proteasome pathway is a rapid way to alter the protein profile and drive cell processes and developmental changes. Many key regulators of embryonic development are targeted for degradation by E3 ubiquitin ligases. The most studied family of E3 ubiquitin ligases is the SCF ubiquitin ligases, which use F-box adaptor proteins to recognize and recruit target proteins. Here, we used a bioinformatics screen and phylogenetic analysis to identify and annotate the family of F-box proteins in the *Xenopus tropicalis* genome. To shed light on the function of the F-box proteins, we analyzed expression of F-box genes during early stages of *Xenopus* development. Many F-box genes are broadly expressed with expression domains localized to diverse tissues including brain, spinal cord, eye, neural crest derivatives, somites, kidneys, and heart. All together, our genome-wide identification and expression profiling of the *Xenopus* F-box family of proteins provide a foundation for future research aimed to identify the precise role of F-box dependent E3 ubiquitin ligases and their targets in the regulatory circuits of development.

## Introduction

Targeted protein ubiquitylation plays fundamental roles in the regulation of multiple cellular processes through proteasome-dependent protein degradation. The majority of this ubiquitylation in eukaryotes is performed by the modular, superfamily of cullin-based E3 ligases [1,2], and the best studied of these E3s are the multi-protein complex SCF (Skp1, Cullin1, F-box) E3 ubiquitin ligases [3–6]. The SCF component Cullin1 serves as a scaffold protein to mediate the transfer of Ubiquitin from the E2 Ub conjugase onto the target protein [7–9]. Transfer of Ub is dependent on three other proteins: Rbx, the RING finger domain protein, which binds to the N-terminus of Cullin1 and recruits Ubiquitin-loaded E2 ubiquitin conjugase; Skp1, which binds to the C-terminus of Cullin1 and the adaptor F-box protein, which facilitates the recruitment of the target protein [2,7,10].

The F-box proteins provide specificity to the SCF complex by binding and recruiting the target proteins via conserved interaction domains with Skp1 and the target proteins [8,10]. The N-terminus of the F-box protein features a 40–50 amino acid “F-box domain” that interacts with the Skp1 protein [10]. The C-terminus of the F-box protein interacts with the target protein and features a variety of protein binding domains including leucine rich repeats (LRR), WD40 repeats, Kelch, between-ring (IBR), and F-box-associated domains (FBA) [11]. Based on the C-terminal substrate binding domain, the F-box proteins are categorized into Fbxw (WD40 (Trp-Asp) repeats), Fbxl (leucine-rich repeats), and Fbxo (other types or no motif) [11].

The diversity of C-terminal target binding domains is critical for the function of the F-box proteins and increases the modularity of the SCF complex, enabling the recognition and recruitment of a large repertoire of target proteins [1,7,12]. Therefore, the family of F-box proteins is large in many eukaryotes: *C*. *elegans* has more than 300, *Arabidopsis* has almost 700, and humans have 70 F-box proteins [11,13,14]. The diversity of F-box proteins is a direct reflection of the functional variety of the SCF ubiquitin ligases [15], and hence they are involved in the regulation of numerous cellular and developmental processes from cell cycle progression [16], to stress response [4,17], to DNA repair [5,6,18], and to neural stem cell maintenance and differentiation [19]. However, only a subset of F-box proteins has been demonstrated to play important roles in embryonic development. Two F-box proteins, Fbxl1 (a.k.a Skp2) and Fbxw7 affect multiple vertebrate developmental processes. Studies in mice show that Fbxl1-mediated degradation of the negative cell cycle regulator p27 is critical for female gamete development [20]. In *Xenopus*, Fbxl1 is expressed in the developing neural plate and brain and negatively regulates neurogenesis to ensure the timely production of neurons [21]. Fbxw7 targets positive regulators of the cell cycle for degradation such as the Notch effector, NICD (Notch intracellular domain) [22–24]. Deletion of Fbxw7 in the mouse brain causes an increase in Notch signaling leading to an increase in neural stem cells and astrocytes at the expense of neurons [25]. Other F-box proteins such as Fbxl14 do not alter the cell cycle but target transcription factors involved in the regulation of neural crest development and the epithelial to mesenchymal transition [26,27].

To expand our understanding of the roles of F-box ubiquitin ligase mediated protein degradation during vertebrate development, we investigated this family in *Xenopus tropicalis* and *X*. *laevis*, two well-characterized model organisms in developmental biology studies. Using phylogenetics, we identified and annotated the family of F-box coding genes in the *X*. *tropicalis* genome. We found that similar to the mammalian genome the *X*. *tropicalis* genome has a large family of F-box proteins with 67 members: 60 mammalian homologs and 7 *Xenopus* specific F-box genes. Expression analysis of the F-box genes revealed that they are transcriptionally active during early developmental stages and expressed dynamically in the embryos. The *Xenopus* F-box genes are expressed in specific organs in the developing embryo including the brain, spinal cord, eyes, neural crest derivatives, somites, kidneys, and heart. Our study provides a perspective to the field of developmental biology from which to further investigate the F-box mediated protein degradation, which may have crucial roles in regulating early embryonic development.

## Materials and Methods

### Bioinformatics and Phylogenetic analysis

The *X*. *tropicalis* genome (version JGI4.2) in the ENSEMBL genome browser [28] was scanned for F-box coding genes using “F-box” as a query statement to retrieve previously annotated genes. Mouse (version GRCm38) and human (version GRCh37) F-box proteins were retrieved from the ENSEMBL genome browser, domain sequences were mapped by Pfam [29] and these were used in a BLAST search analysis [30] to identify *X*. *tropicalis* homologs in the ENSEMBL genome browser. F-box homologs from 5 vertebrate taxa (Puffer fish, Zebrafish, Chicken, Mouse, and Human) were also retrieved from the ENSEMBL genome browser and used for phylogeny construction in S1 Fig. In the Fbxw subfamily phylogeny, the 13 mouse specific Fbxw12-like F-box proteins were not included to manage the tree size.

For phylogenetic analyses, protein sequences were aligned using MAFFT online alignment software [31] with default settings, and alignments were edited with BioEdit [32]. Protein evolutionary models were estimated using ProtTest [33] and the best model was chosen based on–Ln ranking (S1 Table). Maximum likelihood analysis and phylogenetic tree construction were done using PhyML online version [34] with 100 bootstrap replications. Resulting trees were visualized with FigTree v1.4 [35].

For phylogeny of Fbxw7 and Fbw7-like proteins, human, mouse, and *X*. *tropicalis* Fbxw7 proteins and isoforms were aligned with *X*. *tropicalis* putative Fbxw7-like proteins. Sequence and maximum likelihood analyses were as above. Genomic location of each Fbxw7-like gene was determined using ENSEMBL genome browser. Evidence for transcriptional activity of genes and their putative expression domains in frog embryos were gathered from NCBI EST and Unigene [36] databases.

### Reverse Transcription

*X*. *laevis* homologs were identified with BLAST search using *X*. *tropicalis* proteins as query on NCBI EST and non-redundant (nr) nucleotide databases with e-value cut-off = e-10. Out of 67 *Xenopus tropicalis* F-box genes, all but 8 genes (Fbxl4, Fbxo10, Fbxo15, Fbxo32, Fbxo41, Fbxo47, Fbxw10, and Fbxw29) were identified in the *Xenopus laevis* genome. We designed primers for the remaining 59 F-box homologs (S2 Table) in order to study temporal expression with RT-PCR. Total mRNA from egg, early gastrula (st 10.5), neurula (st. 15), and tailbud (st. 25) embryos (10 embryos/stage) was isolated using Ambion RNAqueous RNA isolation kit (Life Sciences Cat. AM1912). The first strand cDNA was synthesized using Moloney Murine Leukemia Virus (M-MMLV) Reverse Transcriptase. To control for genomic DNA contamination, reverse transcription reactions that lacked M-MMLV reverse transcriptase in cDNA synthesis reaction mix (-RT) were used.

### Whole mount in situ hybridization

Whole-mount *in situ* hybridization (WISH) was performed as described [37,38] with the following modifications: pre-hybridization treatment was extended to overnight and an additional 1X SSC wash (15 min, room temperature) was added. To clone genes or fragments for probes, a subset of F-box genes were amplified with PCR. For weakly expressed genes, secondary PCR reactions were performed using the same primers and the products of the first reaction as template. The 20 F-box genes were cloned into pGEMT vector. F-box riboprobes were dioxygenin-labeled.

This study was carried out in strict accordance with the recommendations in the NRC Guide for the Care and Use of Laboratory Animals. The protocol was approved by the Georgetown University Animal Care and Use Committee (GUACUC, Protocol: 13–016). Euthanasia was performed under the American veterinary medical association guidelines, and all efforts were made to minimize suffering.

## Results

### Identification of *Xenopus tropicalis* F-box family of proteins

To study the *Xenopus tropicalis* F-box protein family, we first identified all members of the family by similarity and a simple query search using the *X*. *tropicalis* genome database (genome assembly version 4.2) in ENSEMBLE. We performed a BLASTp search using the 70 human and 82 mouse full-length F-box protein sequences and also searched the *X*. *tropicalis* genome using “F-box” word phrase as query. Furthermore, we performed BLAST searches using all human and mouse F-box domain sequences. With this comprehensive approach, both previously annotated F-box proteins and novel *X*. *tropicalis* specific proteins were retrieved. Our efforts initially identified 105 *Xenopus* F-box homologs. Once redundant sequences and those lacking F-box domains were removed, we identified 67 F-box protein-coding genes (S3 Table).

We annotated the family using phylogenetics with the maximum likelihood approach. The sequences were first sorted manually into three sub-groups according to their target interaction domains (i.e. Fbxl, Fbxo, and Fbxw). To confirm this sorting, the *X*. *tropicalis* sequences were aligned with all three sub-groups of F-box proteins from human, mouse, chick, puffer fish and zebrafish. We found that six *X*. *tropicalis* Fbxo proteins were highly associated (boot strap > 50%) with Fbxl proteins (S1 Fig, yellow shade). The 6 *Xenopus* F-box proteins were, therefore, placed in the LRR subfamily even though the protein sequences lack obvious LRR domains.

**Fig 1:**
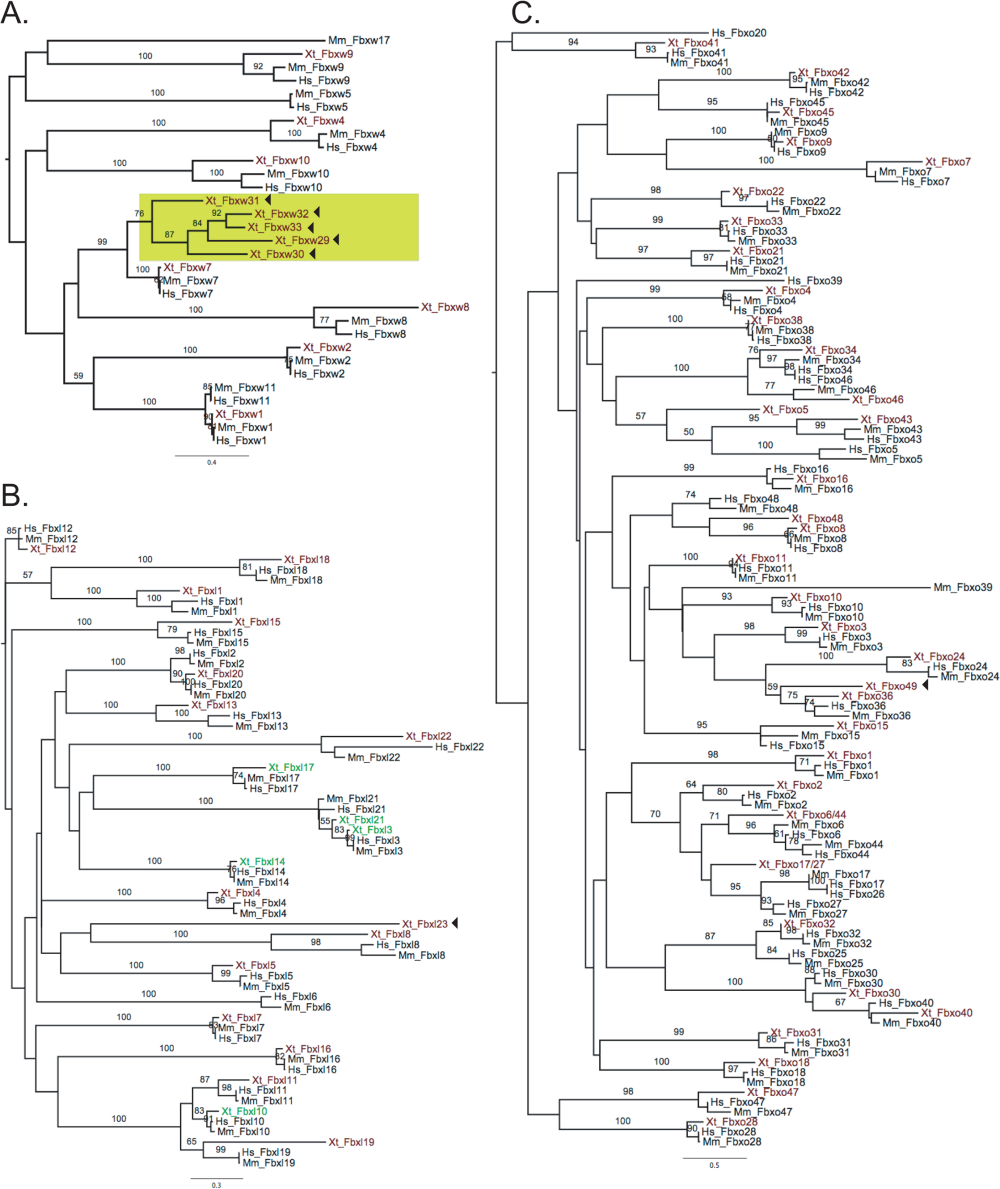
Identification and annotation of *X*. *tropicalis* F-box family of proteins. Full-length protein sequences of F-box proteins categorized into subgroups were used to construct maximum likelihood phylogenies; **A.** F-box proteins with WD40 repeats (Fbxw), **B.** F-box proteins with leucine rich repeats (Fbxl), and **C.** F-box proteins with other domains (Fbxo). A *Xenopus* F-box protein (red) is considered homologous if it clusters with mammalian proteins. Green shaded branch shows *Xenopus* specific Fbxw7-like genes. *Xenopus* Fbxl sequences in green lack an identifiable LRR domain but are found to be highly associated with Fbxl proteins (see S1 Fig). Bootstrap values higher than 50 are shown. Arrowheads show *X*. *tropicalis* specific F-box proteins.

**Fig 2:**
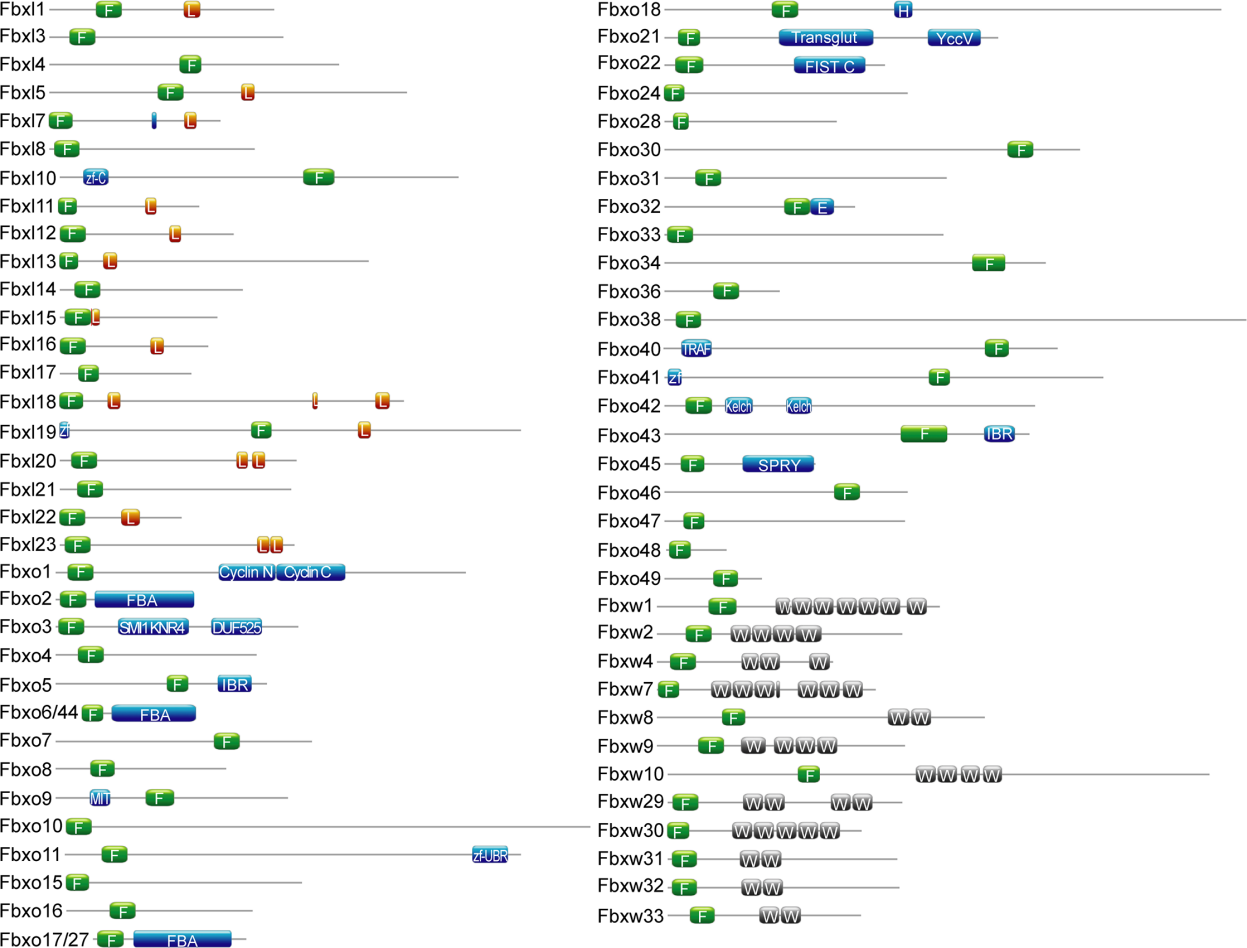
Domain structures of the *Xenopus* F-box proteins. Domains identified by Pfam include F-box motif (F, green), WD40 repeat (WD, gray), leucine-rich repeat (L, orange), F-box-associated domain (FBA), between-ring domain (IBR), Kelch repeat, zf-CXXC zing finger domain (zf-C or zf), Cyclin N and Cyclin C domains, SMI1 KNR4, microtubule interacting and trafficking domain (MIT), zinc finger in N-recognin (Zf-UBR), SPla and the RYanodine Receptor (SPRY), TNF-receptor associated factor (TRAF), Elongin A (E), FIST C domain, Transglutaminase-like superfamily (Transglut), Hemimethylated DNA-binding protein YccV like (YccV), UvrD Helicase (H).

**Fig 3:**
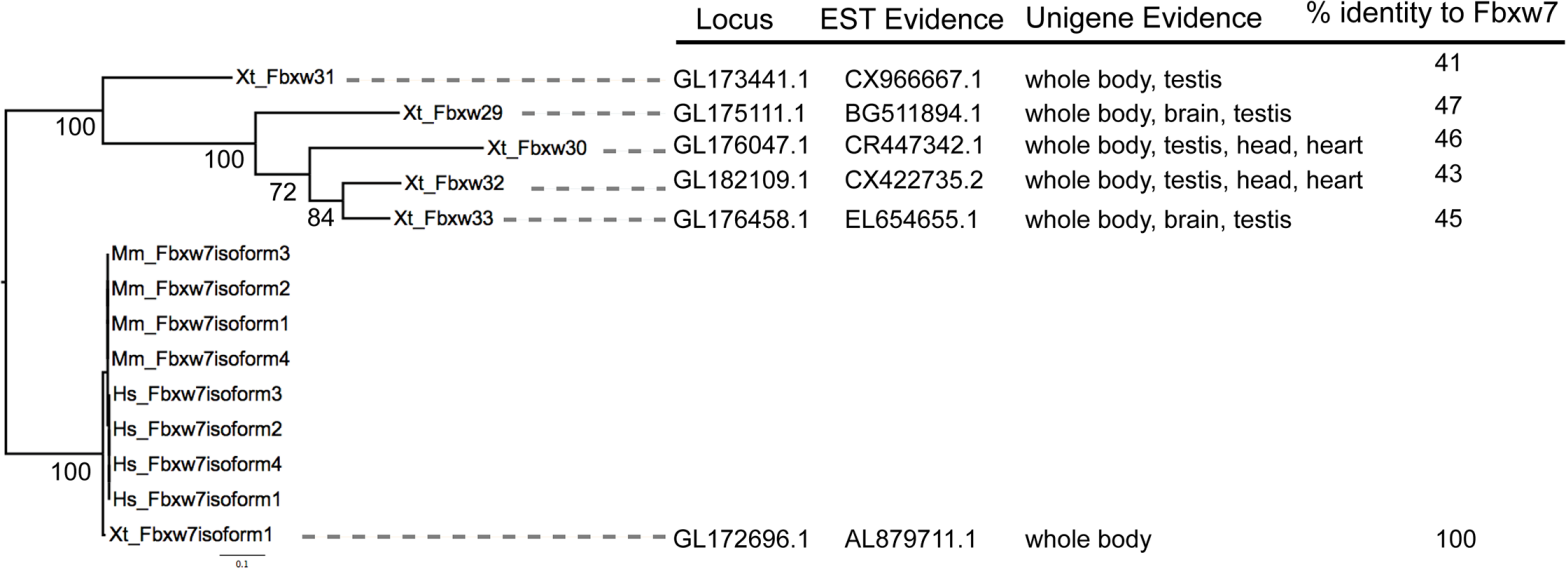
Phylogenetic analysis of five novel *X*. *tropicalis*-specific Fbxw proteins and Fbxw7 isoforms shows that the novel *Xenopus* proteins are similar to Fbxw7. Fbxw7-like genes show moderate similarity to Fbxw7, are in a distinct location in the genome, and expressed in *X*. *tropicalis* (NCBI EST and Unigene databases).

Full-length protein sequences from human, mouse and *Xenopus* were aligned to perform phylogenetic analysis of individual sub-groups (Fbxw, Fbxl or Fbxo). A *Xenopus* F-box protein was considered orthologous when it clustered with mammalian proteins with bootstrap values > 50% (Fig 1). F-box proteins that lack homology were considered novel *Xenopus* F-box proteins. In summary, we identified and annotated 67 putative *Xenopus* F-box protein family members (20 Fbxl, 35 Fbxo, and 12 Fbxw), 7 of which are specific for *Xenopus* (Fig 2). We designated the 7 *Xenopus* specific F-box genes as Fbxl23 (NM_001126660.1), Fbxo49 (NM_001126736.1), Fbxw29 (XP_002944171.2), Fbxw30 (NM_001128022.1), Fbxw31 (NP_001120410.1), Fbxw32 (XM_004913322.1), and Fbxw33 (XM_002944433.2). To confirm that these genes are not pseudogenes, we performed BLAST searches of the NCBI non-redundant protein database. Fbxl23 and Fbxo49 showed little similarity (e-values > 0.01) to other F-box proteins. However, the 5 novel Fbxw genes are similar (e-values < 10^−5^) to Fbxw7. This is also reflected in the phylogenetic trees; the ‘novel’ Fbxw sequences cluster at the Fbxw7 sister clade suggesting that the *X*. *tropicalis* genome has undergone an expansion of Fbxw7-like genes (Fig 1A). To determine if the Fbxw7-like genes are transcribed, we searched the NCBI EST and UNIGENE databases and found evidence for expression at various *X*. *tropicalis* developmental stages and tissues (Fig 3). Furthermore, we determined that they reside in distinct locations in the genome (Fig 3), suggesting that they are not Fbxw7 isoforms. These findings support that *X*. *tropicalis* has expanded Fbxw7-like genes. The expression patterns inferred from EST sources in NCBI database indicate that Fbxl23 is expressed in the head, lung, oviduct, and brain, while Fbxo49 is only expressed in the oviduct. In our analysis, we failed to identify corresponding homologs for 4 mammalian F-box proteins (Fbxl6, Fbxw5, Fbxw11, and Fbxo25) within the *Xenopus* genome. To ensure that these genes were not missed during our scan for F-box homologs, we performed individual BLAST searches on ENSEMBL *X*. *tropicalis* genome and NCBI databases using the 4 mammalian F-box proteins.

### Expression of F-box genes during *X*. *laevis* development

**Fig 4:**
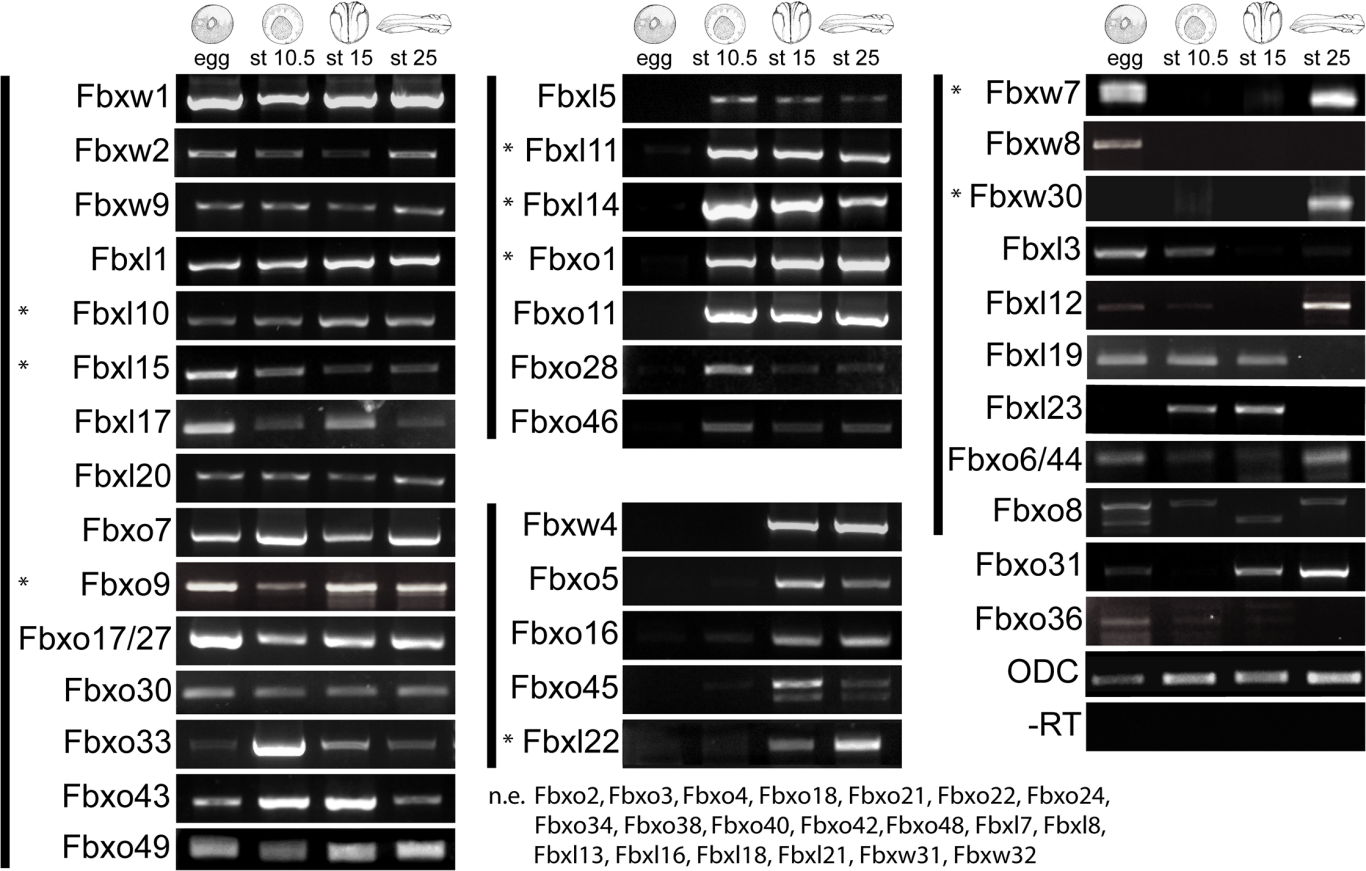
Temporal expression of F-box genes during *X*. *laevis* development. RT-PCR analysis of F-box gene during early embryonic development. ODC was used for loading control.–RT was performed for each gene but a representative for ODC was shown. Expression panels are grouped by shared temporal expression: genes expressed at all 4 stages are in the left column; in 3 zygotic stages in the top middle; in neurula and tailbud in the bottom middle and in a random pattern in the right column. The asterisks indicate the genes whose spatial expressions are shown in Fig 5. N.e. represents genes that are not expressed

To determine if and when the F-box genes are expressed during embryonic development, we performed expression analyses in *X*. *laevis*, which has been the more widely used *Xenopus* species for functional analysis because of its large, robust eggs and rich history in embryological studies. We identified the *X*. *laevis* homologs of the F-box genes with BLASTN searches using *X*. *tropicalis* sequences (S3 Table) and studied the temporal expression of the genes at various developmental stages. We performed RT-PCR analysis on mRNA from egg, early gastrula (stage 10.5) neurula (stage 15) and tailbud embryos (stage 25) (Fig 4, data for 38 F-box genes). We were unable to amplify detect amplified products of Fbxl 7, 8, 13, 16, 18, 21, Fbxo2, 3, 4, 18, 21, 22, 24, 34, 38, 40, 42, 48 and Fbxw31, 32, indicating that these are expressed at very low levels or not expressed during the developmental stages studied. Most of the F-box genes are expressed at low levels and 15 of the genes are expressed at all stages from egg to tailbud (Fig 4, left column), 7 are expressed at all stages except in egg, 5 are predominantly expressed in neurula and tailbud embryos (Fig 4 middle column) and others have a varied expression pattern. For example, Fbxl3 is only expressed until early gastrulation, and Fbxo8 and Fbxo45 primers amplified two bands indicating alternative splice forms. Our temporal expression analyses suggest that more than half (64%) of the F-box genes are active at early developmental stages.

For a complete characterization of the F-box gene family in *X*. *laevis*, we identified the allotetraploid genes for each member of the F-box gene family with a BLASTN search of the genomes using the Xenbase *X*. *laevis* genome browser (*Xenopus laevis* J-strain 8.0 Genome) with an e-value cut off of 1E-30 [39,40]. We retrieved sub-genomic location for each allogene on long (L) and short (S) chromosomes [41,42]. 66 out of 67 *X*. *tropicalis* F-box genes are represented in the *X*. *laevis* sub-genomes (S4 Table). Fbxo47 was the only F-box gene that does not have a homolog in the *X*. *laevis* genome. Next, we determined whether the F-box allogenes are expressed during *X*. *laevis* development by retrieving EST data from the Xenbase expression database for each F-box homolog. Only 2 allogenes, Fbxo5 and Fbxo31, had EST expression data on Xenbase. Combined with our RT-PCR expression analysis, Fbxo5.L is expressed in all four developmental stages studied, while Fbxo5.S is zygotically expressed from stages gastrula to tailbud. Fbxo31.L and Fbxo31.S have similar expression patterns, with both initially expressed maternally followed by a gap in expression in gastrula and resuming expression at neurula and tailbud stages. Future studies focusing on the expression of F-box genes in *X*. *laevis* sub-genomes will clarify the differences in expression of F-box allogenes. Among 66 F-box genes, we retrieved EST expression data of 54 F-box genes including the 2 allogenes from Xenbase. Of the 54 F-box genes, 48 (89%) of the F-box genes have expression overlapping and/or complementary with our expression analysis. There was EST expression data for 3 (6%) F-box genes (Fbxo34, Fbxo38, and Fbxo42) that we were unable to amplify with RT-PCR. In addition to identifying expression to the majority of the *X*. *laevis* F-box genes, our RT-PCR analysis provides expression data to 10 F-box genes (Fbxl12, Fbxl23, Fbxo8, Fbxo16, Fbxo17/27, Fbxo28, Fbxo30, Fbxo36, Fbxo49 and Fbxw30) that did not have any previous EST expression data, 5 of which are novel F-box coding genes (Fbxl23, Fbxo49, Fbxw30, Fbxw31, and Fbxw32). By combining both studies, we found that 20 F-box genes (30%) are expressed at all four developmental stages studied, 26 F-box genes (39%) are maternally expressed while 43 (65%) are zygotically expressed.

**Fig 5:**
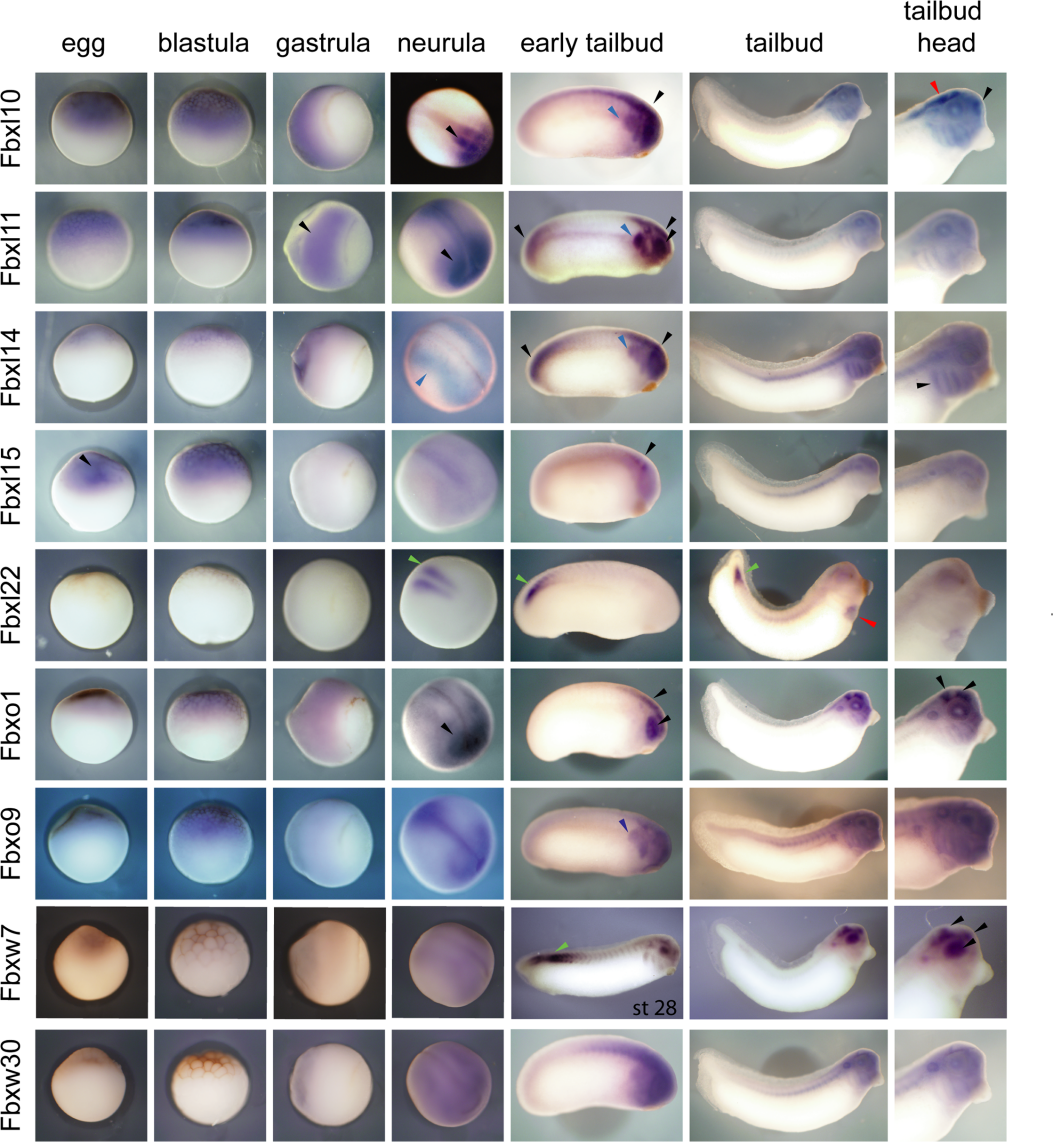
Spatial expression of F-box genes during *X*. *laevis* development using *in situ* hybridizations. Embryos positions: egg and blastula embryos, lateral view with animal side up; gastrula embryos, lateral view with dorsal up and vegetal to the right; neurula embryos dorso-lateral view with anterior to the right; tailbud embryos, lateral view with anterior to the right. Black arrowheads indicate neural tissue, blue—neural crest, green—pre or somitic mesoderm and red–heart.

We examined the spatial expression of a subset of the F-box genes with dynamic temporal expression to uncover their potential functions. Using whole mount *in situ* hybridizations, we analyzed their expression at six developmental stages; egg, blastula (st. 6.5), early gastrula (st. 10.5), neurula (st. 15), early tailbud (st. 20), and tailbud (st. 30) (Fig 5 and S2 Fig). Of 20 F-box genes, 9 showed discrete and dynamic expression during the development of early embryonic tissues (Fig 5) while the rest had diffuse expression (S2 Fig). For example, Fbxl10 expression is restricted to the developing brain of the neurula embryo (black arrowhead) and is also expressed in the neural crest streams in the early tailbud (blue arrowhead). Fbxl11 is restricted to neuroectoderm as early as gastrula stages. The expression is maintained in the neural plate and then in the brain, eye and posterior spinal cord. In addition to the neural tissues, Fbxl11 is also expressed in the n2 and n4 pharyngeal arches at early tailbud stage. However, it is no longer expressed in the neural or the neural crest derivatives in late tailbud embryos. Fbxl14 shows little expression in the egg, blastula, and gastrula embryos; however, it is expressed in the tissues bordering the neural plate, which give rise to neural crest cells. It continues to be expressed in the streaming neural crest and later in all branchial arches in tailbud embryos. The spatial and temporal expression of Fbxl15 expression is highly dynamic throughout development. Transcripts are abundant in the egg and in the animal pole of blastula embryos. However, the expression is lost after the onset of zygotic transcription and is then expressed transiently in the midbrain and hindbrain. Fbxl22 is expressed zygotically and is restricted to the presumptive paraxial mesoderm at neural stages. Later in development, it continues to be expressed in the unsegmented mesoderm of the caudal region at tailbud stages. In older tailbud embryos, it is also expressed in the heart (red arrowhead). Fbxo1 is expressed at all stages of development; however, it is expression is restricted to the anterior neural plate at mid-neurula stage. It is later expressed in midbrain, hindbrain and retina in early tailbud embryos. Fbxo9 is expressed in multiple domains; initially its expression is polarized at the anterior and posterior regions of the neural plate. At early tailbud stage, it is expressed throughout the brain and the migratory neural crest cells. Additionally, at late tailbud stages, it is expressed in pronephric kidney and ducts. Fbxw7 is expressed in the brain, eye and branchial arches of tailbud embryos. It is transiently expressed in the posterior somites and presomitic mesoderm in early tailbud embryos. We identified the *Xenopus* specific Fbxw30 as an Fbxw7-like gene. Although the two genes are phylogenetically related, the expression patterns are quite different. Fbxw30 is not expressed in the mesoderm and is expressed diffusely throughout the head.

## Discussion

Regulation by targeted protein degradation is an underappreciated and yet burgeoning field in developmental biology. Therefore, we characterized the F-box dependent ubiquitin ligase (SCF) family in *X*. *tropicalis* and *X*. *laevis*, two established model systems in developmental biology, towards understanding its role during development. We identified and annotated the 67 member family of F-box coding genes in the *X*. *tropicalis* genome. The expression analyses showed that many of the F-box genes are actively expressed during all developmental stages and their expression is localized to specific organs. These indicate that F-box dependent degradation may be involved in critical developmental events including the development of central nervous system, sensory organs, heart and kidney.

Most of the 70 human and 82 mouse homologs are present in the *Xenopus* genome while 7 F-box genes appear to be species specific. Each vertebrate genome has a similar number of Fbxl and Fbxo subfamilies comprising about 30% and 45–55%, respectively, of the F-box family. However, the percentage of the Fbxw subfamily varies slightly in each genome; 18% and 28% of F-box coding genes in *Xenopus* and mouse genomes belong to Fbxw subfamily, while only 13% are Fbxw in humans. The increase in *Xenopus* and mouse is due to the fact that the Fbxw subfamily underwent an expansion in these genomes. The mouse Fbxw proteins diversified by expansion of Fbxw12-like proteins and in fact, 13 out of 17 Fbxw proteins in mouse are species-specific Fbxw12-like F-box coding genes. A similar expansion is also seen in *Xenopus* Fbxw subfamily; the Fbxw7-like proteins increase the size of the subgroup by 5 related F-box proteins. These indicate that Fbxw subfamily in mouse and frog genomes diversified through expansion of sister Fbxw protein coding genes. The future analysis of the expanded members of the Fbxw subfamily in mouse and frog will help us understand whether the phylogenetic similarity of the proteins are correlated with function and the suite of targets recognized during development of these organisms.

Our report shows that mRNA from 63% of the F-box genes are supplied maternally indicating a crucial role for the induction and specification of germ layers as well as in the coordination of early cell divisions. In addition to the F-box proteins that have established roles in cell cycle regulation including Fbxl1, Fbxw1, and Fbxw7, the suite of F-box genes that have very little known function were also present in the maternal mRNA pool. Future studies will likely identify their precise roles in early embryonic development. The increase in complexity as development proceeds necessitates involvement of additional F-box genes and hence, we show that 87% of the F-box genes that we analyzed were expressed during organogenesis. The expression of a subset of F-box genes in whole embryos revealed restriction of expression to specific organs including the brain, spinal cord, eyes, heart, and kidneys indicating that they specialize in recognition of unique targets that are important for the development of these organs. The F-box genes, even the ones that are known to degrade multiple targets including Fbxl1 and Fbxw7, are expressed at low levels in embryos. This can be due to multiple reasons: (1) low concentrations of F-box proteins are sufficient for degradation, (2) the concentrations of target proteins are low requiring small amounts of F-box protein, and (3) the F-box proteins have a long half-life so that minimal expression is required to maintain the working concentrations in cells.

In summary, we studied an important component of protein degradation machinery in *Xenopus*. The phylogenetics and expressional analysis of the *Xenopus* F-box protein family provide insight into the involvement of targeted protein degradation in the regulation of early embryonic development. Future functional studies will help identify the precise target proteins that are recruited by the F-box proteins and help dismantle the regulatory networks that are involved in development of an embryo.

## Supporting Information

S1 Fig Overall phylogeny of F-box proteins from six vertebra species (*X*. *tropicalis*, puffer fish, zebrafish, chicken, human, and mouse). All members of F-box protein family were used to construct maximum likelihood analysis. *X*. *tropicalis* taxa in red. Branches of the tree were color shaded based on target interaction domains: Fbxl (green), Fbxo (red), and Fbxw (turquoise). The branches with *X*. *tropicalis* F-box sequences that lack identifiable target interaction domains but that cluster with Fbxl subgroup are shaded yellow.
(PNG)

S2 Fig Spatial expression of F-box genes with diffused expression during *X*. *laevis* development using *in situ* hybridizations. Embryos positions: egg and blastula embryos, lateral view with animal side up; gastrula embryos, lateral view with dorsal up and vegetal to the right; neurula embryos dorso-lateral view with anterior to the right; tailbud embryos, lateral view with anterior to the right.
(TIF)

S1 Table The protein evolutionary models used in phylogeny construction estimated by Prottest. (DOCX)

S2 Table Primers used in expressional analysis of the *Xenopus* F-box genes using RT-PCR. (XLSX)

S3 Table Summary of *X*. *tropicalis* F-box family of proteins. (DOCX)

S4 Table Identification of *Xenopus laevis* F-box genes, their genomic locations, and EST expression using Xenbase database. (DOCX)
