# Supplementary figures and images for: Genome-Wide Identification and Expression of Xenopus F-Box Family of Proteins
Source: PLoS One. 2015 Sep 1;10(9):e0136929. doi: 10.1371/journal.pone.0136929 (PMC4556705; doi:10.1371/journal.pone.0136929)

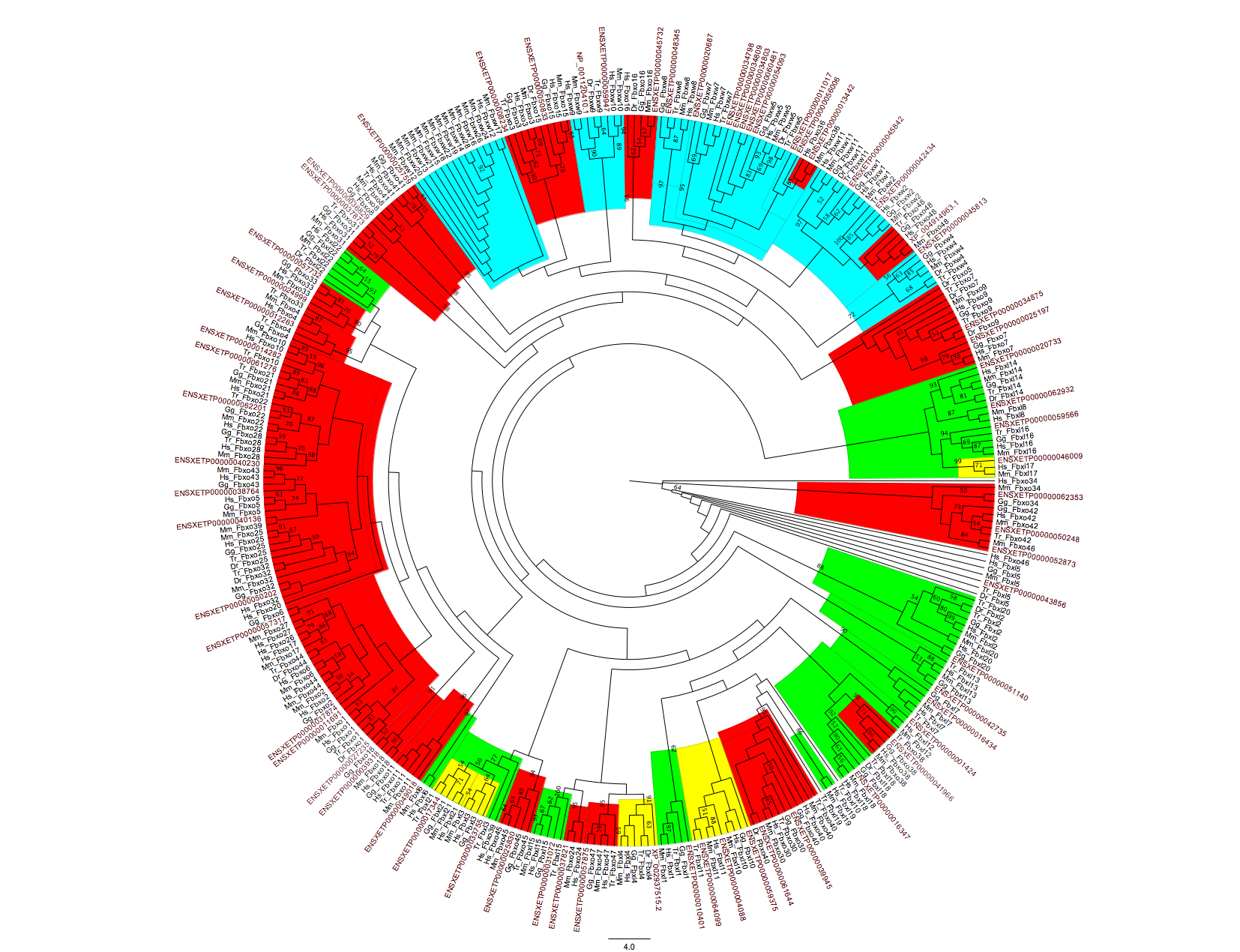

Supplement: S1 Fig — All members of F-box protein family were used to construct maximum likelihood analysis. X. tropicalis taxa in red. Branches of the tree were color shaded based on target interaction domains: Fbxl (green), Fbxo (red), and Fbxw (turquoise). The branches with X. tropicalis F-box sequences that lack identifiable target interaction domains but that cluster with Fbxl subgroup are shaded yellow. (PNG) [file pone.0136929.s001.png]

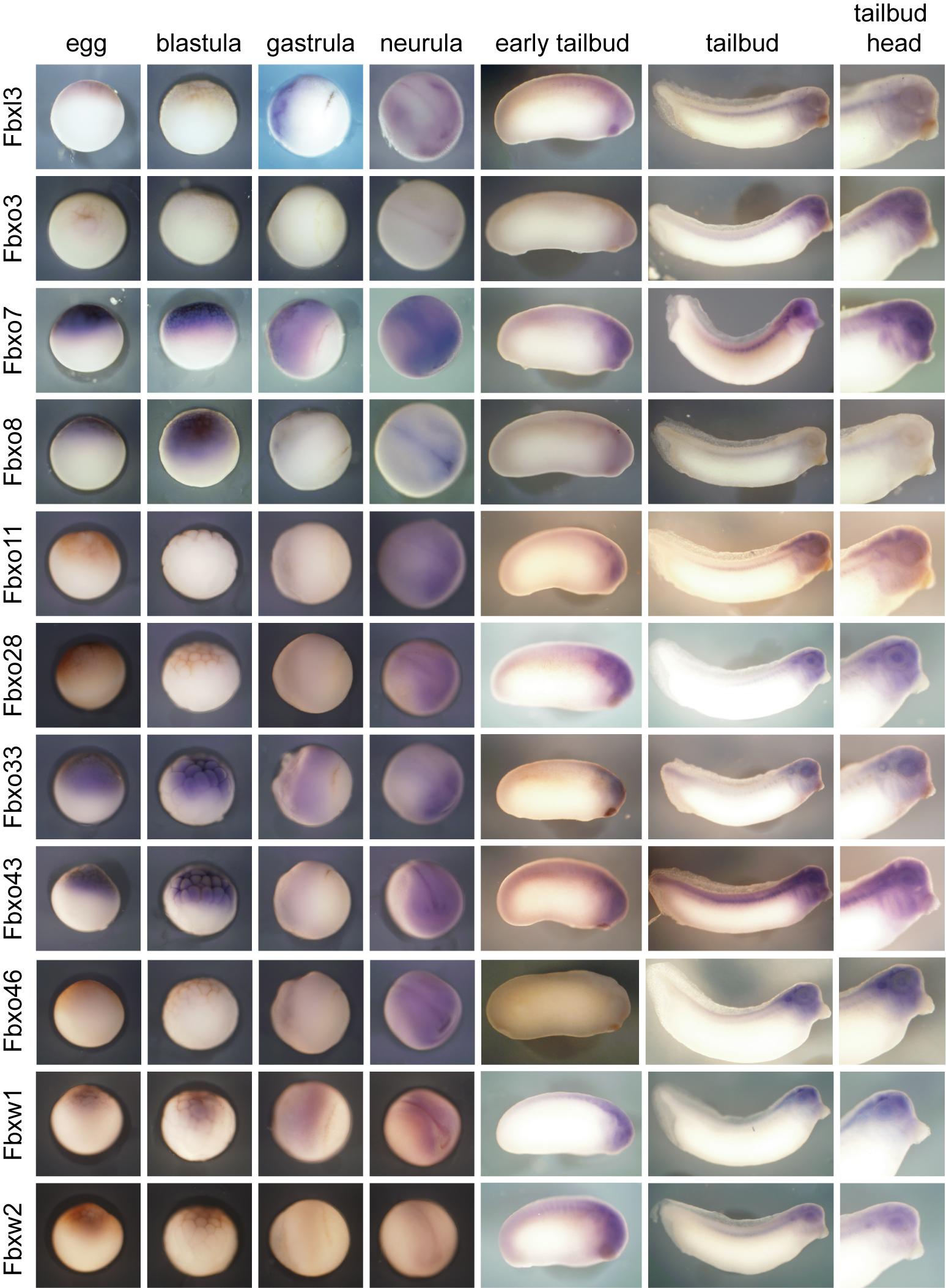

Supplement: S2 Fig — Embryos positions: egg and blastula embryos, lateral view with animal side up; gastrula embryos, lateral view with dorsal up and vegetal to the right; neurula embryos dorso-lateral view with anterior to the right; tailbud embryos, lateral view with anterior to the right. (TIF) [file pone.0136929.s002.tif]
