# Supplementary material for: Genome-Wide Identification and Expression of Xenopus F-Box Family of Proteins
Source: PLoS One. 2015 Sep 1;10(9):e0136929. doi: 10.1371/journal.pone.0136929 (PMC4556705; doi:10.1371/journal.pone.0136929)
Supplement: S1 Table — (DOCX) [file pone.0136929.s003.docx]

**Supplemental Table 1.** The protein evolutionary models used in phylogeny construction were estimated by Prottest.

|  | **Protein Evolutionary Model** | **Gamma Shape Parameter** | **-Ln** |
| --- | --- | --- | --- |
| Fbxl Phylogeny | LG | 3.259 | 14308.05 |
| Fbxo Phylogeny | JTT | 2.473 | 8424.02 |
| Fbxw Phylogeny | WAG | 5.858 | 13226.02 |
| Overall Phylogeny | JTT | 2.848 | 27562.27 |
