# Supplementary material for: Genome-Wide Identification and Expression of Xenopus F-Box Family of Proteins
Source: PLoS One. 2015 Sep 1;10(9):e0136929. doi: 10.1371/journal.pone.0136929 (PMC4556705; doi:10.1371/journal.pone.0136929)
Supplement: S3 Table — (DOCX) [file pone.0136929.s005.docx]

Summary of *X. tropicalis* F-box family of proteins

| **Ensemble Protein ID** | **Annotation** | **Xl Homolog** | **% Similarity** | **Functions** | **Targets** | **Ref.** |
| --- | --- | --- | --- | --- | --- | --- |
| ENSXETP00000010401 | Fbxl1 | DY563494.1 NM_001096366 | 88 | Oncogene, neural differantiation, panreatic cancer, cell cycle regulation, vascular smooth muscle cell proliferation, hepatocellular carcinoma, viral lytic replication, adipogenesis, regulation of chromatin modifiers, beta cell regeneration in pancreas, neuroblastoma, thyroid cancer, lung cancer, breast cancer, transcriptional elongation, DNA recombination, TGF-beta signaling, cervical cancer, DNA repair, notch signaling | Prohibitin, Inhibitor of growth family member 3 (ING3), p21 (3-refs), p27, p57, p130, Foxo1, RINGO/Speedy A, Tumor suppressor TIS21, Cyclin G2, Sp1, Cyclin A, SET8, p300,Tal1/SCL, MLL, Tob1, MEF/ELK4, Xic1, RASSF1A, c-myc, Retinoblastoma protein, RAG-2,ISG15 Isopeptidase UBP43,SMAD4,Papillomavirus E7 Oncoprotein, Cdt1, E2A, CDK9/cyclin T1 complex, Cyclin E, BRCA1 | [1–77] |
| ENSXETP00000033755 | Fbxl3 | EB735959.1 NM_001093513 | 94 | Circadian clock | Cry1, Cry2, Sofia | [78–82] |
| XP_002937515.2 | Fbxl4 | N/A | N/A | mtDNA maintenance | N.d. | [83,84] |
| ENSXETP00000043856 | Fbxl5 | EB478649.1 NM_001091592 | 90 | Iron metabolism, vesicular trafficking and transport, endometrial carcinoma | Iron regulatory proteins 2 (IRP2), p150 (Glued) | [85–89] |
| ENSXETP00000016434 | Fbxl7 | DY569381.1 | 92 | Breast cancer | N.d. | [90] |
| ENSXETP00000062932 | Fbxl8 | BC106573 | 90 | N.d. | N.d. |  |
| ENSXETP00000059375 | Fbxl10 | EG581744.1 NM_001092371 | 91 | Gene silencing, transcription repressor, cellular lifespan, stem cell maintenance, repression of rRNA genes, neural development | BCOR, c-fos promoter, c-Jun, c-Jun promoter, rDNA | [91–97] |
| ENSXETP00000064099 | Fbxl11 | CA987939.1 NM_001173992 | 94 | Histone demethylase, regulation of rRNA transcription, regulation of NF-kB | N.d. | [98–100] |
| ENSXETP00000001424 | Fbxl12 | KC506729 | 84 | N.d. | N.d. |  |
| ENSXETP00000042735 | Fbxl13 | CD302831.1 | 96 | Candidate myeloid tumor suppressor | N.d. | [101] |
| ENSXETP00000020733 | Fbxl14 | BX849893.1 NM_001090376 | 90 | Epithelial mesenchymal transition, neural crest development | Slug, Snail, Twist, Sip1 | [102–104] |
| ENSXETP00000031072 | Fbxl15 | EB476395.1 NM_001086278 | 89 | BMP signaling | Smurf1 | [105] |
| ENSXETP00000033877 | Fbxl16 | EB474372.1 | 91 | Cell proliferation | N.d. | [106] |
| ENSXETP00000046009 | Fbxl17 | DR716016.1 NM_001127772 | 85 | Breast cancer | N.d. | [107] |
| ENSXETP00000016347 | Fbxl18 | EB645757 | 92 | N.d. | N.d. |  |
| ENSXETP00000004088 | Fbxl19 | CA987939.1 NM_001173992 | 91 | Psoriasis arthiritis | N.d. | [108] |
| ENSXETP00000051140 | Fbxl20 | CD328092.1 NM_001093596 | 88 | Fear memory formation, synaptic transmission | Rim1 | [109,110] |
| ENSXETP00000017344 | Fbxl21 | NP_001086982.1 | 81 | Circadian clock, schizophrenia | Cry1 | [79,111] |
| ENSXETP00000021454 | Fbxl22 | BJ032069.1  NM_001096890 | 87 | Cardiomyocyte homeostatis | Alpha-actinin, Filamin C | [112] |
| ENSXETP00000057732 | Fbxl23* | CA982418.1 NM_001096136 | 92 | Novel | N.d. |  |
| ENSXETP00000027235 | Fbxo1 | BX843736.1 | 91 | Cell cycle regulation, centrosome homeostasis | CP110, CyclinB1 | [113–118] |
| ENSXETP00000037874 | Fbxo2 | CF547522.1 | 90 | Regulation of malformed glycoproteins, cochlear homeostasis, viral infection maintenance of neurons | RNAse B, Lactoferrin, HSV-1-encoded UL9 protein, NR1 subunit of NMDA receptor | [119–123] |
| ENSXETP00000008234 | Fbxo3 | NM_001093708 | 90 | PML-dependent transcriptional regulation | HIPK2 | [124] |
| ENSXETP00000012263 | Fbxo4 | NM_001127741 | 87 | Regulation of cell cycle telomere maintenance | CyclinD1, PIN2/TRF1 | [125–128] |
| ENSXETP00000040136 | Fbxo5 | CB207903.1 | 89 | Regulation of cell cycle, breast cancer | BRCA1 | [129–131] |
| ENSXETP00000057317 | Fbxo6/44 | BM192559.1 | 89 | Endoplasmic reticulum associated degradation of gycoproteins, DNA replication, gastric cancer, breast cancer | ErolL, TFRC, RPN1, Erlin 2, SMC1, SMC3, Chk1, Lactoferrin, Heparin, BRCA1 | [131–135] |
| ENSXETP00000025197 | Fbxo7 | CX131255 | 85 | Neurodegeneration, Parkinson's disease, apoptosis, NFkB signaling, cell cycle regulation | CIAP1, CyclinD, Cdk6, p27 |  |
| ENSXETP00000036829 | Fbxo8 | CD254327.1 | 90 | N.d. | N.d. |  |
| ENSXETP00000034875 | Fbxo9 | NM_001087129 | 93 | Innate immunity | N.d. |  |
| ENSXETP00000014282 | Fbxo10 | N/A | N/A | Breast cancer | N.d. |  |
| ENSXETP00000049018 | Fbxo11 | BU917012.1  NM_001093135 | 94 | Otitis media, TGF-beta pathway, p53 neddylation, melanoctye formation, heterochrony, arginine methytransferase, tumor suppressor | p53, BCL6 |  |
| ENSXETP00000050833 | Fbxo15 | N/A | N/A | Embryonic stem cell maintenance | N.d. |  |
| ENSXETP00000045732 | Fbxo16 | CX131969.1 | 85 | N.d. | N.d. |  |
| ENSXETP00000011691 | Fbxo17/27 | CF547374 | 89 | Degradation of glycoproteins | Lactoferrin, Heparin |  |
| ENSXETP00000039318 | Fbxo18 | CF520902.1 | 88 | Helicase/nucleic acid metabolism | N.d. |  |
| ENSXETP00000061276 | Fbxo21 | BU910290 | 94 | N.d. | N.d. |  |
| ENSXETP00000062201 | Fbxo22 | BJ642492.1 | 92 | Pathogenesis, histone modification | GogB, Kdm4A |  |
| ENSXETP00000037821 | Fbxo24 | CV074022.1 | 83 | N.d. | N.d. |  |
| ENSXETP00000040230 | Fbxo28 | BX854965.1 | 93 | N.d. | N.d. |  |
| ENSXETP00000039945 | Fbxo30 | CA789318.1 | 88 | N.d. | N.d. |  |
| ENSXETP00000037873 | Fbxo31 | BQ732841.1 | 89 | DNA damage response, tumor suppressor | CyclinD1 |  |
| ENSXETP00000050202 | Fbxo32 | N/A | 91 | Gastric cancer, regulation of muscle wasting | SerpinB5, MyoD, Calcineurin, eIF3-f |  |
| ENSXETP00000024999 | Fbxo33 | BU913032.1 | 90 | Programmed cell death, seizure | Y-box binding protein 1 (YB-1)/dbpB/p50 |  |
| ENSXETP00000062353 | Fbxo34 | NM_001097840 | 88 | N.d. | N.d. |  |
| ENSXETP00000013442 | Fbxo36 | AW645510.1 | N/A | N.d. | N.d. |  |
| ENSXETP00000041966 | Fbxo38 | BI444638.1 | 93 | N.d. | N.d. |  |
| ENSXETP00000061644 | Fbxo40 | CB560117.1 | 88 | IGF1 signaling in skeletal muscle, muscle dystrophy | IRS1 |  |
| ENSXETP00000025759 | Fbxo41 | N/A | N/A | N.d. | N.d. |  |
| ENSXETP00000050248 | Fbxo42 | BU907399.1 | 86 | Putative role in congenital cataract | N.d. |  |
| ENSXETP00000038764 | Fbxo43 | BU914013.1 | 88 | Embryonic pre-MBT divisions, meiotic arrest | Cdc20, Cdc27, Anaphase-promoting complex/cyclosome (APC/C) |  |
| ENSXETP00000025830 | Fbxo45 | BX844092.1 | 92 | Synapse formation /neurotransmission, cell cycle arrest and apoptosis, neuronal development | Munc13, p73 |  |
| ENSXETP00000052873 | Fbxo46 | NM_001098714 | 91 | N.d. | N.d. |  |
| ENSXETP00000057875 | Fbxo47 | N/A | N/A | Putative tumor suppressor in papillary renal cell carcinoma | N.d. |  |
| XP_004914963.1 | Fbxo48 | DT065657 | 100 | N.d | N.d |  |
| ENSXETP00000056006 | Fbxo49* | EB644343 | 91 | Novel | N.d. |  |
| ENSXETP00000045842 | Fbxw1 | BQ383755.1 | 96 | Cancer, retina development, DNA repair, oxidative stress, mitosis, neural differentiation, chromosome stability, decidualization, TGF-beta signaling, Hedgehog signaling, Wnt signaling, HIV, circadian clock, cellular stress response, immune and inflammatory response, axis formation, cell cycle regulation | Mdm2, FOXO3, Fanconi anemia (FA) M protein, RCAN1, BORA, REST, Epithelium-specific ETS transcription factor-1 (ESE-1), SMAD4, Gli2, Gli3, Prolactin receptor, PER1, FGD1 Cdc42 Guanine nucleotide exchange factor, Beta-catenin, Emi1, NFkB1, p105, ATF4, IkBalpha, IkBbeta-epsilon, HIV-1 Vpu protein, CDC25B |  |
| ENSXETP00000042434 | Fbxw2 | BQ734235.1 | 89 | Glial cell differentiation, trophoblast cell differentiation | GCMa |  |
| ENSXETP00000045813 | Fbxw4 | BU914556 | 93 | Distal limb development | N.d. |  |
| ENSXETP00000020687 | Fbxw7 | AW640941.1 | 95 | Cell cycle regulation, tumor suppressor, TGF-beta signaling, p63, adipogenesis suppressor, neural crest development, hematopoiesis, cholesterol and lipid metabolism, notch signaling, apoptosis, vascular development, Alzheimer’s disease, synaptogenesis, sex determination | TGIF, C/EBP alpha, Klf5, Proto-oncoprotein c-Myb, SREBP1, Cyclin E1 and E2, mTOR, p18-cyclin E, c-Jun, c-myc, Notch ICD, Presenilin1, Sel-12 Preselinin, FEM proteins |  |
| ENSXETP00000048345 | Fbxw8 | NM_001092209 | 91 | Negative regulator of oncogenesis, placental development | BCL-3 |  |
| NP_001120410.1 | Fbxw9 | NM_001095104 | 91 | N.d. | N.d. |  |
| ENSXETP00000059947 | Fbxw10 | N/A | 90 | Lamina assembly | N.d. |  |
| ENSXETP00000011017 | Fbxw29* | AB467369.1 | 98 | Novel | N.d |  |
| ENSXETP00000034803 | Fbxw30* | BQ733700 NM_001096074 | 93 | Novel | N.d |  |
| NP_001121494.1 | Fbxw31* | NP_001089543.1 | 83 | Novel | N.d. |  |
| ENSXETP00000034798 | Fbxw32* | NM_001096074 | 83 | Novel | N.d. |  |
| ENSXETP00000054093 | Fbxw33* | NP_001089543.1 | 81 | Novel | N.d. |  |

*X. laevis* homologs were identified with BLASTP search on NCBI database. Literature search was performed on Pubmed database using F-box protein name (e.g. Fbxl1) as query. A protein was considered an F-box target when direct interaction evidence such as co-immunoprecipitation or X-ray crystallography was present. Novel proteins are marked with asterisks. N/A, Not Available; N.d., Not Determined.

**References for Supplemental Table 3**

1. Ang XL, Harper JW (2004) Interwoven ubiquitination oscillators and control of cell cycle transitions. Sci STKE 2004: pe31. doi:10.1126/stke.2422004pe31.

2. Barboric M, Zhang F, Besenicar M, Plemenitas A, Peterlin BM (2005) Ubiquitylation of Cdk9 by Skp2 facilitates optimal Tat transactivation. J Virol 79: 11135. doi:10.1128/JVI.79.17.11135.

3. Ben-Izhak O, Akrish S, Gan S, Nagler RM (2009) Skp2 and salivary cancer. Cancer Biol Ther 8: 153–158.

4. Bendjennat M, Boulaire J, Jascur T, Brickner H, Barbier V, et al. (2003) UV irradiation triggers ubiquitin-dependent degradation of p21(WAF1) to promote DNA repair. Cell 114: 599–610.

5. Bhattacharya S, Garriga J, Calbó J, Yong T, Haines DS, et al. (2003) SKP2 associates with p130 and accelerates p130 ubiquitylation and degradation in human cells. Oncogene 22: 2443–2451. doi:10.1038/sj.onc.1206339.

6. Boix-Perales H, Horan I, Wise H, Lin H-R, Chuang L-C, et al. (2007) The E3 ubiquitin ligase skp2 regulates neural differentiation independent from the cell cycle. Neural Dev 2: 27. doi:10.1186/1749-8104-2-27.

7. Calvisi DF, Ladu S, Pinna F, Frau M, Tomasi ML, et al. (2009) SKP2 and CKS1 promote degradation of cell cycle regulators and are associated with hepatocellular carcinoma prognosis. Gastroenterology 137: 1816–1826.e1–e10. doi:10.1053/j.gastro.2009.08.005.

8. Calvisi DF, Pinna F, Ladu S, Muroni MR, Frau M, et al. (2010) The degradation of cell cycle regulators by SKP2/CKS1 ubiquitin ligase is genetically controlled in rodent liver cancer and contributes to determine the susceptibility to the disease. Int J Cancer 126: 1275–1281. doi:10.1002/ijc.24650.

9. Carrano a C, Eytan E, Hershko a, Pagano M (1999) SKP2 is required for ubiquitin-mediated degradation of the CDK inhibitor p27. Nat Cell Biol 1: 193–199.

10. Chander H, Halpern M, Resnick-Silverman L, Manfredi JJ, Germain D (2010) Skp2B attenuates p53 function by inhibiting prohibitin. EMBO Rep 11: 220–225. doi:10.1038/embor.2010.2.

11. Chen G, Wang Y, Garate M, Zhou J, Li G (2010) The tumor suppressor ING3 is degraded by SCF(Skp2)-mediated ubiquitin-proteasome system. Oncogene 29: 1498–1508. doi:10.1038/onc.2009.424.

12. Chen J-Y, Wang M-C, Hung W-C (2009) Transcriptional activation of Skp2 by BCR-ABL in K562 chronic myeloid leukemia cells. Leuk Res 33: 1520–1524. doi:10.1016/j.leukres.2009.03.007.

13. Chiappetta G, De Marco C, Quintiero A, Califano D, Gherardi S, et al. (2007) Overexpression of the S-phase kinase-associated protein 2 in thyroid cancer. Endocr Relat Cancer 14: 405–420. doi:10.1677/ERC-06-0030.

14. Cooke PS, Holsberger DR, Cimafranca MA, Meling DD, Beals CM, et al. (2007) Cellular and Molecular The F Box Protein S Phase Kinase-associated Protein 2 Regulates Adipose Mass and Adipocyte Number in Vivo. Obesity 15: 1400–1408.

15. Dardente H, Mendoza J, Fustin J-M, Challet E, Hazlerigg DG (2008) Implication of the F-Box Protein FBXL21 in circadian pacemaker function in mammals. PLoS One 3: e3530. doi:10.1371/journal.pone.0003530.

16. Dinarina A, Santamaria PG, Nebreda AR (2009) Cell cycle regulation of the mammalian CDK activator RINGO/Speedy A. FEBS Lett 583: 2772–2778. doi:10.1016/j.febslet.2009.07.028.

17. Ecker K, Hengst L (2009) Skp2: caught in the Akt. Nat Cell Biol 11: 377–379. doi:10.1038/ncb1859.

18. Fotovati A, Abu-Ali S, Nakayama K, Nakayama KI (2011) Impaired ovarian development and reduced fertility in female mice deficient in Skp2. J Anat 218: 668–677. doi:10.1111/j.1469-7580.2011.01370.x.

19. Gstaiger M, Jordan R, Lim M, Catzavelos C, Mestan J, et al. (2001) Skp2 is oncogenic and overexpressed in human cancers. Proc Natl Acad Sci U S A 98: 5043–5048. doi:10.1073/pnas.081474898.

20. Harmey D, Smith A, Simanski S, Moussa CZ, Ayad NG (2009) The anaphase promoting complex induces substrate degradation during neuronal differentiation. J Biol Chem 284: 4317–4323. doi:10.1074/jbc.M804944200.

21. Hershko DD (2008) Oncogenic properties and prognostic implications of the ubiquitin ligase Skp2 in cancer. Cancer 112: 1415–1424. doi:10.1002/cncr.23317.

22. Hiramatsu Y, Kitagawa K, Suzuki T, Uchida C, Hattori T, et al. (2006) Degradation of Tob1 mediated by SCFSkp2-dependent ubiquitination. Cancer Res 66: 8477–8483. doi:10.1158/0008-5472.CAN-06-1603.

23. Huang H, Regan KM, Wang F, Wang D, Smith DI, et al. (2005) Skp2 inhibits FOXO1 in tumor suppression through ubiquitin-mediated degradation. Proc Natl Acad Sci U S A 102: 1649–1654. doi:10.1073/pnas.0406789102.

24. Iwahori S, Murata T, Kudoh A, Sato Y, Nakayama S, et al. (2009) Phosphorylation of p27Kip1 by Epstein-Barr virus protein kinase induces its degradation through SCFSkp2 ubiquitin ligase actions during viral lytic replication. J Biol Chem 284: 18923–18931. doi:10.1074/jbc.M109.015123.

25. Jiang H, Chang F-C, Ross AE, Lee J, Nakayama K, et al. (2005) Ubiquitylation of RAG-2 by Skp2-SCF links destruction of the V(D)J recombinase to the cell cycle. Mol Cell 18: 699–709. doi:10.1016/j.molcel.2005.05.011.

26. Kalra N, Kumar V (2006) The X protein of hepatitis B virus binds to the F box protein Skp2 and inhibits the ubiquitination and proteasomal degradation of c-Myc. FEBS Lett 580: 431–436. doi:10.1016/j.febslet.2005.12.034.

27. Kamura T, Hara T, Kotoshiba S, Yada M, Ishida N, et al. (2003) Degradation of p57Kip2 mediated by SCFSkp2-dependent ubiquitylation. Proc Natl Acad Sci U S A 100: 10231–10236. doi:10.1073/pnas.1831009100.

28. Kiernan RE, Emiliani S, Nakayama K, Castro A, Labbe JC, et al. (2001) Interaction between cyclin T1 and SCFSKP2 targets CDK9 for ubiquitination and degradation by the proteasome. Mol Cell Biol 21: 7956–7970. doi:10.1128/MCB.21.23.7956.

29. Kim SY, Herbst A, Tworkowski KA, Salghetti SE, Tansey WP (2003) Skp2 Regulates Myc Protein Stability and Activity. Mol Cell 11: 1177–1188.

30. Kitagawa K, Kotake Y, Kitagawa M (2009) Ubiquitin-mediated control of oncogene and tumor suppressor gene products. Cancer Sci 100: 1374–1381. doi:10.1111/j.1349-7006.2009.01196.x.

31. Kitagawa M, Lee SH, McCormick F (2008) Skp2 suppresses p53-dependent apoptosis by inhibiting p300. Mol Cell 29: 217–231. doi:10.1016/j.molcel.2007.11.036.

32. Knight JS, Sharma N, Robertson ES (2005) Epstein-Barr virus latent antigen 3C can mediate the degradation of the retinoblastoma protein through an SCF cellular ubiquitin ligase. Proc Natl Acad Sci U S A 102: 18562–18566. doi:10.1073/pnas.0503886102.

33. Kondo T, Kobayashi M, Tanaka J, Yokoyama A, Suzuki S, et al. (2004) Rapid degradation of Cdt1 upon UV-induced DNA damage is mediated by SCFSkp2 complex. J Biol Chem 279: 27315–27319. doi:10.1074/jbc.M314023200.

34. Li X, Zhao Q, Liao R, Sun P, Wu X (2003) The SCF(Skp2) ubiquitin ligase complex interacts with the human replication licensing factor Cdt1 and regulates Cdt1 degradation. J Biol Chem 278: 30854–30858. doi:10.1074/jbc.C300251200.

35. Liang M, Liang YY, Wrighton K, Ungermannova D, Wang XP, et al. (2004) Ubiquitination and proteolysis of cancer-derived Smad4 mutants by SCFSkp2. Mol Cell Biol 24: 7524–7537. doi:10.1128/MCB.24.17.7524.

36. Lin H, Chuang L, Boix-perales H, Philpott A, Yew R (2006) Ubiquitination of Cylclin-Dependent Kinase Inhibitor, Xic1, is mediated by the Xenopus F-box Protein xSkp2. Cell Cycle 5: 304–314.

37. Liu H, Cheng EH-Y, Hsieh JJ-D (2007) Bimodal degradation of MLL by SCFSkp2 and APCCdc20 assures cell cycle execution: a critical regulatory circuit lost in leukemogenic MLL fusions. Genes Dev 21: 2385–2398. doi:10.1101/gad.1574507.

38. Liu Y, Hedvat C V, Mao S, Zhu X, Yao J, et al. (2006) The ETS protein MEF is regulated by phosphorylation-dependent proteolysis via the protein-ubiquitin ligase SCFSkp2. Mol Cell Biol 26: 3114–3123. doi:10.1128/MCB.26.8.3114.

39. Nakayama K, Nagahama H, Minamishima Y a, Miyake S, Ishida N, et al. (2004) Skp2-mediated degradation of p27 regulates progression into mitosis. Dev Cell 6: 661–672.

40. Nie L, Wu H, Sun X-H (2008) Ubiquitination and degradation of Tal1/SCL are induced by notch signaling and depend on Skp2 and CHIP. J Biol Chem 283: 684–692. doi:10.1074/jbc.M704981200.

41. Nie L, Xu M, Vladimirova A, Sun X-H (2003) Notch-induced E2A ubiquitination and degradation are controlled by MAP kinase activities. EMBO J 22: 5780–5792. doi:10.1093/emboj/cdg567.

42. Nishitani H, Sugimoto N, Roukos V, Nakanishi Y, Saijo M, et al. (2006) Two E3 ubiquitin ligases, SCF-Skp2 and DDB1-Cul4, target human Cdt1 for proteolysis. EMBO J 25: 1126–1136. doi:10.1038/sj.emboj.7601002.

43. Oh KJ, Kalinina A, Wang J, Nakayama K, Nakayama KI, et al. (2004) The papillomavirus E7 oncoprotein is ubiquitinated by UbcH7 and Cullin 1-and Skp2-containing E3 ligase. J Virol 78: 5338–5346. doi:10.1128/JVI.78.10.5338.

44. Okada M, Sakai T, Nakamura T, Tamamori-Adachi M, Kitajima S, et al. (2009) Skp2 promotes adipocyte differentiation via a p27Kip1-independent mechanism in primary mouse embryonic fibroblasts. Biochem Biophys Res Commun 379: 249–254. doi:10.1016/j.bbrc.2008.12.069.

45. Park TJ, Kim JY, Park SH, Kim HS, Lim IK (2009) Skp2 enhances polyubiquitination and degradation of TIS21/BTG2/PC3, tumor suppressor protein, at the downstream of FoxM1. Exp Cell Res 315: 3152–3162. doi:10.1016/j.yexcr.2009.07.009.

46. Radke S, Pirkmaier A, Germain D (2005) Differential expression of the F-box proteins Skp2 and Skp2B in breast cancer. Oncogene 24: 3448–3458. doi:10.1038/sj.onc.1208328.

47. Rodriguez S, Wang L, Mumaw C, Srour EF, Lo Celso C, et al. (2011) The SKP2 E3 ligase regulates basal homeostasis and stress-induced regeneration of HSCs. Blood 117: 6509–6519. doi:10.1182/blood-2010-11-321521.

48. Rosner M, Hanneder M, Siegel N, Valli a, Fuchs C, et al. (2009) Skp2 inversely correlates with p27 and tuberin in transformed cells. Amino Acids 37: 257–262. doi:10.1007/s00726-008-0141-7.

49. Sakai T, Sakaue H, Nakamura T, Okada M, Matsuki Y, et al. (2007) Skp2 controls adipocyte proliferation during the development of obesity. J Biol Chem 282: 2038–2046. doi:10.1074/jbc.M608144200.

50. Salon C, Merdzhanova G, Brambilla C, Brambilla E, Gazzeri S, et al. (2007) E2F-1, Skp2 and cyclin E oncoproteins are upregulated and directly correlated in high-grade neuroendocrine lung tumors. Oncogene 26: 6927–6936. doi:10.1038/sj.onc.1210499.

51. Sarmento LM, Huang H, Limon A, Gordon W, Fernandes J, et al. (2005) Notch1 modulates timing of G1-S progression by inducing SKP2 transcription and p27 Kip1 degradation. J Exp Med 202: 157–168. doi:10.1084/jem.20050559.

52. Schuler S, Diersch S, Hamacher R, Schmid RM, Saur D, et al. (2011) SKP2 confers resistance of pancreatic cancer cells towards TRAIL-induced apoptosis. Int J Oncol 38: 219. doi:10.3892/ijo.

53. Song MS, Song SJ, Kim SJ, Nakayama K, Nakayama KI, et al. (2008) Skp2 regulates the antiproliferative function of the tumor suppressor RASSF1A via ubiquitin-mediated degradation at the G1-S transition. Oncogene 27: 3176–3185. doi:10.1038/sj.onc.1210971.

54. Sonoda H, Inoue H, Ogawa K, Utsunomiya T, Masuda T, et al. (2006) Significance of skp2 expression in primary breast cancer. Clin Cancer Res 12: 1215–1220. doi:10.1158/1078-0432.CCR-05-1709.

55. Suzuki S, Fukasawa H, Misaki T, Togawa A, Ohashi N, et al. (2011) Up-regulation of Cks1 and Skp2 with TNFα/NF-κB signaling in chronic progressive nephropathy. Genes Cells 16: 1110–1120. doi:10.1111/j.1365-2443.2011.01553.x.

56. Tang Y, Simoneau AR, Liao W, Yi G, Hope C, et al. (2009) WIF1, a Wnt pathway inhibitor, regulates SKP2 and c-myc expression leading to G1 arrest and growth inhibition of human invasive urinary bladder cancer cells. Mol Cancer Ther 8: 458–468. doi:10.1158/1535-7163.MCT-08-0885.

57. Tapias A, others (2008) Regulation of Sp1 by cell cycle related proteins. Cell Cycle 7: 2856–2867.

58. Tokarz S, Berset C, La Rue J, Friedman K, Nakayama K-I, et al. (2004) The ISG15 isopeptidase UBP43 is regulated by proteolysis via the SCFSkp2 ubiquitin ligase. J Biol Chem 279: 46424–46430. doi:10.1074/jbc.M403189200.

59. Tschen S-I, Georgia S, Dhawan S, Bhushan A (2011) Skp2 Is Required for Incretin Hormone-Mediated {beta}-Cell Proliferation. Mol Endocrinol 25: 2134–2143. doi:10.1210/me.2011-1119.

60. Von der Lehr N, Johansson S, Wu S, Bahram F, Castell A, et al. (2003) The F-box protein Skp2 participates in c-Myc proteosomal degradation and acts as a cofactor for c-Myc-regulated transcription. Mol Cell 11: 1189–1200.

61. Wang F, Chan C-H, Chen K, Guan X, Lin H-K, et al. (2011) Deacetylation of FOXO3 by SIRT1 or SIRT2 leads to Skp2-mediated FOXO3 ubiquitination and degradation. Oncogene: 1–12. doi:10.1038/onc.2011.347.

62. Wang J, Han F, Wu J, Lee S-W, Chan C-H, et al. (2011) The role of Skp2 in hematopoietic stem cell quiescence, pool size and self-renewal. Blood: 5429–5438. doi:10.1182/blood-2010-10-312785.

63. Wang W, Nacusi L, Sheaff RJ, Liu X (2005) Ubiquitination of p21Cip1/WAF1 by SCFSkp2: substrate requirement and ubiquitination site selection. Biochemistry 44: 14553–14564. doi:10.1021/bi051071j.

64. Wang X-C, Wu Y-P, Ye B, Lin D-C, Feng Y-B, et al. (2009) Suppression of anoikis by SKP2 amplification and overexpression promotes metastasis of esophageal squamous cell carcinoma. Mol Cancer Res 7: 12–22. doi:10.1158/1541-7786.MCR-08-0092.

65. Ward EC, Hoekstra A V, Blok LJ, Hanifi-Moghaddam P, Lurain JR, et al. (2008) The regulation and function of the forkhead transcription factor, Forkhead box O1, is dependent on the progesterone receptor in endometrial carcinoma. Endocrinology 149: 1942–1950. doi:10.1210/en.2007-0756.

66. Wei W, Ayad NG, Wan Y, Zhang G-J, Kirschner MW, et al. (2004) Degradation of the SCF component Skp2 in cell-cycle phase G1 by the anaphase-promoting complex. Nature 428: 194–198. doi:10.1038/nature02381.

67. Westermann F, Henrich K-O, Wei JS, Lutz W, Fischer M, et al. (2007) High Skp2 expression characterizes high-risk neuroblastomas independent of MYCN status. Clin Cancer Res 13: 4695–4703. doi:10.1158/1078-0432.CCR-06-2818.

68. Wu W, Sun X-H (2011) A mechanism underlying notch-induced and ubiquitin-mediated Jak3 degradation. J Biol Chem 286: 41153–41162. doi:10.1074/jbc.M111.273755.

69. Wu Y-J, Sala-Newby GB, Shu K-T, Yeh H-I, Nakayama KI, et al. (2009) S-phase kinase-associated protein-2 (Skp2) promotes vascular smooth muscle cell proliferation and neointima formation in vivo. J Vasc Surg  Off Publ Soc Vasc Surg [and] Int Soc Cardiovasc Surgery, North Am Chapter 50: 1135–1142. doi:10.1016/j.jvs.2009.07.066.

70. Xu G, Bernaudo S, Fu G, Lee DY, Yang BB, et al. (2008) Cyclin G2 is degraded through the ubiquitin-proteasome pathway and mediates the antiproliferative effect of activin receptor-like kinase 7. Mol Biol Cell 19: 4968. doi:10.1091/mbc.E08.

71. Xu S, Abbasian M, Patel P, Jensen-Pergakes K, Lombardo CR, et al. (2007) Substrate recognition and ubiquitination of SCFSkp2/Cks1 ubiquitin-protein isopeptide ligase. J Biol Chem 282: 15462–15470. doi:10.1074/jbc.M610758200.

72. Yam CH, Ng RW, Siu WY, Lau a W, Poon RY (1999) Regulation of cyclin A-Cdk2 by SCF component Skp1 and F-box protein Skp2. Mol Cell Biol 19: 635–645.

73. Yam CH, Siu WY, Lau a, Poon RY (2000) Degradation of cyclin A does not require its phosphorylation by CDC2 and cyclin-dependent kinase 2. J Biol Chem 275: 3158–3167.

74. Yang Y, Chen KY, Tong Q (2011) Murine Sirt3 protein isoforms have variable half-lives. Gene 488: 46–51. doi:10.1016/j.gene.2011.07.029.

75. Yeh KH, Kondo T, Zheng J, Tsvetkov LM, Blair J, et al. (2001) The F-box protein SKP2 binds to the phosphorylated threonine 380 in cyclin E and regulates ubiquitin-dependent degradation of cyclin E. Biochem Biophys Res Commun 281: 884–890. doi:10.1006/bbrc.2001.4442.

76. Yin Y, Yu VC, Zhu G, Chang DC (2008) SET8 plays a role in controlling G1/S transition by blocking lysine acetylation in histone through binding to H4 N-terminal tail. Cell Cycle 7: 1423–1432.

77. Zhong L, Georgia S, Tschen S, Nakayama K, Nakayama K, et al. (2007) Essential role of Skp2-mediated p27 degradation in growth and adaptive expansion of pancreatic beta cells. J Clin Invest 117: 2869–2876. doi:10.1172/JCI32198.ubiquitin-proteosome.

78. Busino L, Bassermann F, Maiolica A, Lee C, Nolan PM, et al. (2007) SCFFbxl3 controls the oscillation of the circadian clock by directing the degradation of cryptochrome proteins. Science 316: 900–904. doi:10.1126/science.1141194.

79. Dardente H, Mendoza J, Fustin J-M, Challet E, Hazlerigg DG (2008) Implication of the F-Box Protein FBXL21 in circadian pacemaker function in mammals. PLoS One 3: e3530. doi:10.1371/journal.pone.0003530.

80. Godinho SIH, Maywood ES, Shaw L, Tucci V, Barnard AR, et al. (2007) The after-hours mutant reveals a role for Fbxl3 in determining mammalian circadian period. Science 316: 897–900. doi:10.1126/science.1141138.

81. Maywood ES, Chesham JE, Meng Q-J, Nolan PM, Loudon ASI, et al. (2011) Tuning the period of the mammalian circadian clock: additive and independent effects of CK1εTau and Fbxl3Afh mutations on mouse circadian behavior and molecular pacemaking. J Neurosci 31: 1539–1544. doi:10.1523/JNEUROSCI.4107-10.2011.

82. Siepka SM, Yoo S-H, Park J, Song W, Kumar V, et al. (2007) Circadian mutant Overtime reveals F-box protein FBXL3 regulation of cryptochrome and period gene expression. Cell 129: 1011–1023. doi:10.1016/j.cell.2007.04.030.

83. Bonnen PE, Yarham JW, Besse A, Wu P, Faqeih E a, et al. (2013) Mutations in FBXL4 cause mitochondrial encephalopathy and a disorder of mitochondrial DNA maintenance. Am J Hum Genet 93: 471–481. doi:10.1016/j.ajhg.2013.07.017.

84. Gai X, Ghezzi D, Johnson M a, Biagosch C a, Shamseldin HE, et al. (2013) Mutations in FBXL4, encoding a mitochondrial protein, cause early-onset mitochondrial encephalomyopathy. Am J Hum Genet 93: 482–495. doi:10.1016/j.ajhg.2013.07.016.

85. Salahudeen A a, Thompson JW, Ruiz JC, Ma H-W, Kinch LN, et al. (2009) An E3 ligase possessing an iron-responsive hemerythrin domain is a regulator of iron homeostasis. Science 326: 722–726. doi:10.1126/science.1176326.

86. Rouault T a (2009) Cell biology. An ancient gauge for iron. Science 326: 676–677. doi:10.1126/science.1181938.

87. Vashisht A a, Zumbrennen KB, Huang X, Powers DN, Durazo A, et al. (2009) Control of iron homeostasis by an iron-regulated ubiquitin ligase. Science 326: 718–721. doi:10.1126/science.1176333.

88. Zhang N, Liu J, Ding X, Aikhionbare F, Jin C, et al. (2007) FBXL5 interacts with p150Glued and regulates its ubiquitination. Biochem Biophys Res Commun 359: 34–39. doi:10.1016/j.bbrc.2007.05.068.

89. Moroishi T, Nishiyama M, Takeda Y, Iwai K, Nakayama KI (2011) The FBXL5-IRP2 Axis Is Integral to Control of Iron Metabolism In Vivo. Cell Metab 14: 339–351. doi:10.1016/j.cmet.2011.07.011.

90. Wang X, Pankratz VS, Fredericksen Z, Tarrell R, Karaus M, et al. (2010) Common variants associated with breast cancer in genome-wide association studies are modifiers of breast cancer risk in BRCA1 and BRCA2 mutation carriers. Hum Mol Genet 19: 2886–2897. doi:10.1093/hmg/ddq174.

91. Tzatsos A, Paskaleva P, Lymperi S, Contino G, Stoykova S, et al. (2011) A Lysine (K)-specific demethylase 2B (KDM2B)-let-7-Enhancer of Zester Homolog 2 (EZH2) pathway regulates cell cycle progression and senescence in primary cells. J Biol Chem 286: 33061–33069. doi:10.1074/jbc.M111.257667.

92. Ge R, Wang Z, Zeng Q, Xu X, Olumi a F (2011) F-box protein 10, an NF-κB-dependent anti-apoptotic protein, regulates TRAIL-induced apoptosis through modulating c-Fos/c-FLIP pathway. Cell Death Differ 18: 1184–1195. doi:10.1038/cdd.2010.185.

93. Fukuda T, Tokunaga A, Sakamoto R, Yoshida N (2011) Fbxl10/Kdm2b deficiency accelerates neural progenitor cell death and leads to exencephaly. Mol Cell Neurosci 46: 614–624. doi:10.1016/j.mcn.2011.01.001.

94. Konuma T, Nakamura S, Miyagi S, Negishi M, Chiba T, et al. (2011) Forced expression of the histone demethylase Fbxl10 maintains self-renewing hematopoietic stem cells. Exp Hematol 39: 697–709.e5. doi:10.1016/j.exphem.2011.03.008.

95. Frescas D, Guardavaccaro D, Bassermann F, Koyama-Nasu R, Pagano M (2007) JHDM1B/FBXL10 is a nucleolar protein that represses transcription of ribosomal RNA genes. Nature 450: 309–313. doi:10.1038/nature06255.

96. Gearhart MD, Corcoran CM, Wamstad J a, Bardwell VJ (2006) Polycomb group and SCF ubiquitin ligases are found in a novel BCOR complex that is recruited to BCL6 targets. Mol Cell Biol 26: 6880–6889. doi:10.1128/MCB.00630-06.

97. Koyama-Nasu R, David G, Tanese N (2007) The F-box protein Fbl10 is a novel transcriptional repressor of c-Jun. Nat Cell Biol 9: 1074–1080. doi:10.1038/ncb1628.

98. Tanaka Y, Okamoto K, Teye K, Umata T, Yamagiwa N, et al. (2010) JmjC enzyme KDM2A is a regulator of rRNA transcription in response to starvation. EMBO J 29: 1510–1522. doi:10.1038/emboj.2010.56.

99. Lu T, Jackson MW, Wang B, Yang M, Chance MR, et al. (2010) Regulation of NF-kappaB by NSD1/FBXL11-dependent reversible lysine methylation of p65. Proc Natl Acad Sci U S A 107: 46–51. doi:10.1073/pnas.0912493107.

100. Lu T, Jackson MW, Singhi AD, Kandel ES, Yang M, et al. (2009) Validation-based insertional mutagenesis identifies lysine demethylase FBXL11 as a negative regulator of NFkappaB. Proc Natl Acad Sci U S A 106: 16339–16344. doi:10.1073/pnas.0908560106.

101. Curtiss NP, Bonifas JM, Lauchle JO, Balkman JD, Kratz CP, et al. (2005) Isolation and analysis of candidate myeloid tumor suppressor genes from a commonly deleted segment of 7q22. Genomics 85: 600–607. doi:10.1016/j.ygeno.2005.01.013.

102. Vernon AE, LaBonne C (2006) Slug stability is dynamically regulated during neural crest development by the F-box protein Ppa. Development 133: 3359–3370. doi:10.1242/dev.02504.

103. Lander R, Nordin K, LaBonne C (2011) The F-box protein Ppa is a common regulator of core EMT factors Twist, Snail, Slug, and Sip1. J Cell Biol 194: 17–25. doi:10.1083/jcb.201012085.

104. Viñas-Castells R, Beltran M, Valls G, Gómez I, García JM, et al. (2010) The hypoxia-controlled FBXL14 ubiquitin ligase targets SNAIL1 for proteasome degradation. J Biol Chem 285: 3794–3805. doi:10.1074/jbc.M109.065995.

105. Cui Y, He S, Xing C, Lu K, Wang J, et al. (2011) SCFFBXL^15^ regulates BMP signalling by directing the degradation of HECT-type ubiquitin ligase Smurf1. EMBO J 30: 2675–2689. doi:10.1038/emboj.2011.155.

106. Sato K, Kusama Y, Tategu M, Yoshida K (2010) FBXL16 is a novel E2F1-regulated gene commonly upregulated in p16INK4A-and p14ARF-silenced HeLa cells. Int J Oncol 36: 479–490. doi:10.3892/ijo.

107. Xiao GG, Zhou B-S, Somlo G, Portnow J, Juhasz A, et al. (2008) Identification of F-box/LLR-repeated protein 17 as potential useful biomarker for breast cancer therapy. Cancer Genomics Proteomics 5: 151–160.

108. Stuart PE, Nair RP, Ellinghaus E, Ding J, Tejasvi T, et al. (2010) Genome-wide association analysis identifies three psoriasis susceptibility loci. Nat Genet 42: 1000–1004. doi:10.1038/ng.693.

109. Yao I, Takagi H, Ageta H, Kahyo T, Sato S, et al. (2007) SCRAPPER-dependent ubiquitination of active zone protein RIM1 regulates synaptic vesicle release. Cell 130: 943–957. doi:10.1016/j.cell.2007.06.052.

110. Yao I, Takao K, Miyakawa T, Ito S, Setou M (2011) Synaptic E3 ligase SCRAPPER in contextual fear conditioning: extensive behavioral phenotyping of Scrapper heterozygote and overexpressing mutant mice. PLoS One 6: e17317. doi:10.1371/journal.pone.0017317.

111. Chen X, Wang X, Sun C, Chen Q, O’Neill F, et al. (2008) FBXL21 association with schizophrenia in irish family and case–control samples. Am J Med Genet Part B Neuropsychiatr Genet 147B: 1231–1237.

112. Spaich S, Will RD, Just S, Spaich S, Kuhn C, et al. (2012) Fbxl22, A Cardiac-Enriched F-Box Protein, Regulates Sarcomeric Protein Turnover and is Essential for Maintenance of Contractile Function In Vivo. Circ Res. doi:10.1161/CIRCRESAHA.112.271007.

113. Tetzlaff M, Bai C, Finegold M (2004) Cyclin F Disruption Compromises Placental Development and Affects Normal Cell Cycle. … Cell Biol 24: 2487–2498. doi:10.1128/MCB.24.6.2487.

114. Fung TK, Siu WY, Yam CH, Lau A, Poon RYC (2002) Cyclin F is degraded during G2-M by mechanisms fundamentally different from other cyclins. J Biol Chem 277: 35140–35149. doi:10.1074/jbc.M205503200.

115. Kong M, Barnes E a, Ollendorff V, Donoghue DJ (2000) Cyclin F regulates the nuclear localization of cyclin B1 through a cyclin-cyclin interaction. EMBO J 19: 1378–1388. doi:10.1093/emboj/19.6.1378.

116. Emanuele MJ, Elia AEH, Xu Q, Thoma CR, Izhar L, et al. (2011) Global identification of modular cullin-RING ligase substrates. Cell 147: 459–474. doi:10.1016/j.cell.2011.09.019.

117. Bai C, Richman R, Elledge S (1994) Human cyclin F. EMBO J 13: 6087–6098.

118. D’Angiolella V, Donato V, Vijayakumar S, Saraf A, Florens L, et al. (2010) SCF(Cyclin F) controls centrosome homeostasis and mitotic fidelity through CP110 degradation. Nature 466: 138–142. doi:10.1038/nature09140.

119. Erhardt J a, Hynicka W, DiBenedetto a, Shen N, Stone N, et al. (1998) A novel F box protein, NFB42, is highly enriched in neurons and induces growth arrest. J Biol Chem 273: 35222–35227.

120. Glenn K a, Nelson RF, Wen HM, Mallinger AJ, Paulson HL (2008) Diversity in tissue expression, substrate binding, and SCF complex formation for a lectin family of ubiquitin ligases. J Biol Chem 283: 12717–12729. doi:10.1074/jbc.M709508200.

121. Yamaguchi Y, Hirao T, Sakata E, Kamiya Y, Kurimoto E, et al. (2007) Fbs1 protects the malfolded glycoproteins from the attack of peptide:N-glycanase. Biochem Biophys Res Commun 362: 712–716. doi:10.1016/j.bbrc.2007.08.056.

122. Nelson RF, Glenn K a, Zhang Y, Wen H, Knutson T, et al. (2007) Selective cochlear degeneration in mice lacking the F-box protein, Fbx2, a glycoprotein-specific ubiquitin ligase subunit. J Neurosci 27: 5163–5171. doi:10.1523/JNEUROSCI.0206-07.2007.

123. Eom C-Y, Heo W Do, Craske ML, Meyer T, Lehman IR (2004) The neural F-box protein NFB42 mediates the nuclear export of the herpes simplex virus type 1 replication initiator protein (UL9 protein) after viral infection. Proc Natl Acad Sci U S A 101: 4036–4040. doi:10.1073/pnas.0400738101.

124. Shima Y, Shima T, Chiba T, Irimura T, Pandolfi PP, et al. (2008) PML activates transcription by protecting HIPK2 and p300 from SCFFbx3-mediated degradation. Mol Cell Biol 28: 7126–7138. doi:10.1128/MCB.00897-08.

125. Jia L, Sun Y (2009) F-box proteins FBXO31 and FBX4 in regulation of cyclin D1 degradation upon DNA damage. Pigment Cell Melanoma Res 22: 518–519. doi:10.1111/j.1755-148X.2009.00611.x.

126. Lin DI, Barbash O, Kumar KGS, Weber JD, Harper JW, et al. (2006) Phosphorylation-dependent ubiquitination of cyclin D1 by the SCF(FBX4-alphaB crystallin) complex. Mol Cell 24: 355–366. doi:10.1016/j.molcel.2006.09.007.

127. Li Y, Hao B (2010) Structural basis of dimerization-dependent ubiquitination by the SCF(Fbx4) ubiquitin ligase. J Biol Chem 285: 13896–13906. doi:10.1074/jbc.M110.111518.

128. Lee TH, Perrem K, Harper JW, Lu KP, Zhou XZ (2006) The F-box protein FBX4 targets PIN2/TRF1 for ubiquitin-mediated degradation and regulates telomere maintenance. J Biol Chem 281: 759–768. doi:10.1074/jbc.M509855200.

129. Chen J-Y, Wang M-C, Hung W-C (2011) Bcr-Abl-induced tyrosine phosphorylation of Emi1 to stabilize Skp2 protein via inhibition of ubiquitination in chronic myeloid leukemia cells. J Cell Physiol 226: 407–413. doi:10.1002/jcp.22346.

130. Di Fiore B, Pines J (2007) Emi1 is needed to couple DNA replication with mitosis but does not regulate activation of the mitotic APC/C. J Cell Biol 177: 425–437. doi:10.1083/jcb.200611166.

131. Lu Y, Li J, Cheng D, Parameswaran B, Zhang S, et al. (2012) The F-box protein FBXO44 mediates BRCA1 ubiquitination and degradation. J Biol Chem: 1–21. doi:10.1074/jbc.M112.407106.

132. Zhang L, Hou Y, Wang M, Wu B, Li N (2009) A study on the functions of ubiquitin metabolic system related gene FBG2 in gastric cancer cell line. J Exp Clin Cancer Res 28: 78. doi:10.1186/1756-9966-28-78.

133. Glenn K a, Nelson RF, Wen HM, Mallinger AJ, Paulson HL (2008) Diversity in tissue expression, substrate binding, and SCF complex formation for a lectin family of ubiquitin ligases. J Biol Chem 283: 12717–12729. doi:10.1074/jbc.M709508200.

134. Liu B, Zheng Y, Wang T-D, Xu H-Z, Xia L, et al. (2012) Proteomic identification of common SCF ubiquitin ligase FBXO6-interacting glycoproteins in three kinds of cells. J Proteome Res 11: 1773–1781. doi:10.1021/pr2010204.

135. Zhang Y-W, Brognard J, Coughlin C, You Z, Dolled-Filhart M, et al. (2009) The F box protein Fbx6 regulates Chk1 stability and cellular sensitivity to replication stress. Mol Cell 35: 442–453. doi:10.1016/j.molcel.2009.06.030.
