# Supplementary material for: Genome-Wide Identification and Expression of Xenopus F-Box Family of Proteins
Source: PLoS One. 2015 Sep 1;10(9):e0136929. doi: 10.1371/journal.pone.0136929 (PMC4556705; doi:10.1371/journal.pone.0136929)
Supplement: S4 Table — (DOCX) [file pone.0136929.s006.docx]

**S4 Table:** Identification of *Xenopus laevis* F-box genes, their genomic locations, and EST expression using Xenbase database. *X. tropicalis* F-box genes were used to BLAST the *X. laevis* genome database (e-value cut off: 1E-30). EST expression data from Xenbase was retrieved and compared to our RT-PCR data. Color legend is at the end. The F-box genes with expression data, followed by the F-box allogenes with unknown expression, are shown below. Only two F-box genes, Fbxo5 and Fbxo31 (in bold), were identified with expression in both allotetraploid genes. The genomic location is indicated by *X. laevis* chromosome number with long (L) or short (S) version of the chromosome followed by gene start and end nucleotides. F-box genes with sub-genome locations are indicated by a suffix of either “.L” or “.S” in gene names.

| **Xl Genomic Location** | **F-box gene** | **e-value** | **egg** | **gastrula** | **neurula** | **tailbud** |
| --- | --- | --- | --- | --- | --- | --- |
| XLv80.chr1L:193658851-193659109 | Fbxl1 | 1E-127 |  |  |  |  |
| XLv80.chr2L:110558187-110559251 | Fbxl3 | 0 |  |  |  |  |
| XLv80.chr7L:86511521-86512014 | Fbxl4.L | 0 |  |  |  |  |
| XLv80.chr1L:19548438-19549123 | Fbxl5.L | 0 |  |  |  |  |
| XLv80.Sc006756_chrNA:1476-2512 | Fbxl7 | 0 |  |  |  |  |
| XLv80.chr4L:40151515-40152566 | Fbxl8 | 0 |  |  |  |  |
| XLv80.chr1L:154275837-154276858 | Fbxl10 | 0 |  |  |  |  |
| XLv80.chr7L:59493445-59494497 | Fbxl11 | 0 |  |  |  |  |
| XLv80.chr3L:122041580-122042479 | Fbxl12 | 0 |  |  |  |  |
| XLv80.chr3S:2888364-2889755 | Fbxl14 | 0 |  |  |  |  |
| XLv80.chr3L:62641871-62642123 | Fbxl13 | 6E-85 |  |  |  |  |
| XLv80.chr7L:39183436-39184090 | Fbxl15.a | 0 |  |  |  |  |
| XLv80.Sc000190_chrNA:30505-32195 | Fbxl16 | 0 |  |  |  |  |
| XLv80.chr1L:167895390-167896587 | Fbxl17 | 0 |  |  |  |  |
| XLv80.chr9_10L:85456976-85458534 | Fbxl18 | 0 |  |  |  |  |
| XLv80.Sc000164_chrNA:50370-51004 | Fbxl19 | 0 |  |  |  |  |
| XLv80.chr9_10L:2397849-2398644 | Fbxl20.L | 0 |  |  |  |  |
| XLv80.chr3L:7220946-7221589 | Fbxl21 | 0 |  |  |  |  |
| XLv80.chr3S:57182320-57183525 | Fbxl22.S | 0 |  |  |  |  |
| XLv80.chr8S:15764609-15765336 | Fbxl23* | 0 |  |  |  |  |
| XLv80.chr9_10L:104633301-104633761 | Fbxo1 | 0 |  |  |  |  |
| XLv80.chr7S:45965141-45965394 | Fbxo2 | 1E-88 |  |  |  |  |
| XLv80.chr4S:15863911-15864723 | Fbxo3 | 0 |  |  |  |  |
| XLv80.chr1L:191139613-191140526 | Fbxo4.L | 0 |  |  |  |  |
| XLv80.chr5S:37508581-37509267 | **Fbxo5.S** | 0 |  |  |  |  |
| XLv80.chr5L:47781153-47781854 | **Fbxo5.L** | 0 |  |  |  |  |
| XLv80.chr7S:45998015-45998242 | Fbxo6/44 | 3E-88 |  |  |  |  |
| XLv80.Sc000028_chrNA:1218005-1218864 | Fbxo7.a | 0 |  |  |  |  |
| XLv80.chr1L:40790706-40791314 | Fbxo8.b | 0 |  |  |  |  |
| XLv80.chr5L:94161486-94161966 | Fbxo9.L | 0 |  |  |  |  |
| XLv80.chr1S:93197999-93198843 | Fbxo10 | 0 |  |  |  |  |
| XLv80.chr5L:20913572-20913791 | Fbxo11 | 8E-108 |  |  |  |  |
| XLv80.chr6S:71066286-71066583 | Fbxo15 | 6E-115 |  |  |  |  |
| XLv80.chr5L:154027357-154027567 | Fbxo16 | 2E-70 |  |  |  |  |
| XLv80.chr8S:83485001-83485394 | Fbxo17/27 | 0 |  |  |  |  |
| XLv80.chr3L:68934003-68934513 | Fbxo18 | 4e-162 |  |  |  |  |
| XLv80.chr1L:157145490-157145746 | Fbxo21 | 1E-92 |  |  |  |  |
| XLv80.chr3L:74480123-74480816 | Fbxo22 | 0 |  |  |  |  |
| XLv80.chr3L:135019192-135019474 | Fbxo24 | 5.E-71 |  |  |  |  |
| XLv80.chr5S:12796770-12796955 | Fbxo28.S | 4E-90 |  |  |  |  |
| XLv80.chr5L:40675463-40677244 | Fbxo30 | 0 |  |  |  |  |
| XLv80.chr4S:31851343-31851815 | **Fbxo31.S** | 0 |  |  |  |  |
| XLv80.chr4L:54059803-54060271 | **Fbxo31.L** | 0 |  |  |  |  |
| XLv80.chr8L:69647882-69648569 | Fbxo33.L | 0 |  |  |  |  |
| XLv80.chr8L:84059139-84060734 | Fbxo34.L | 0 |  |  |  |  |
| XLv80.chr5S:111170711-111171028 | Fbxo36 | 6E-115 |  |  |  |  |
| XLv80.chr3L:30782968-30783213 | Fbxo38 | 6E-122 |  |  |  |  |
| XLv80.chr2L:2304861-2306609 | Fbxo40 | 0 |  |  |  |  |
| XLv80.chr1L:810693-811679 | Fbxo41 | 0 |  |  |  |  |
| XLv80.Sc002489_chrNA:1-585 | Fbxo42.L | 0 |  |  |  |  |
| XLv80.chr6L:98979109-98980547 | Fbxo43.L | 0 |  |  |  |  |
| XLv80.chr5S:111721706-111722961 | Fbxo45.b | 0 |  |  |  |  |
| XLv80.chr8L:50832059-50833573 | Fbxo46.a | 0 |  |  |  |  |
| XLv80.chr5L:28183938-28184166 | Fbxo48 | 8E-74 |  |  |  |  |
| XLv80.chr2L:95154604-95154776 | Fbxo49* | 4E-61 |  |  |  |  |
| XLv80.chr7S:19705380-19706180 | Fbxw1 | 0 |  |  |  |  |
| XLv80.chr8L:5091244-5091760 | Fbxw2 | 0 |  |  |  |  |
| XLv80.chr7L:25705512-25706910 | Fbxw4 | 0 |  |  |  |  |
| XLv80.chr1L:48503417-48503929 | Fbxw7.L | 0 |  |  |  |  |
| XLv80.chr1L:157075359-157075960 | Fbxw8.L | 0 |  |  |  |  |
| XLv80.chr3S:119259868-119260782 | Fbxw9.b | 0 |  |  |  |  |
| XLv80.chr4S:6019815-6020775 | Fbxw30* | 0 |  |  |  |  |
| XLv80.chr4S:6019472-6021025 | Fbxw31* | 0 |  |  |  |  |
| XLv80.chr4L:984819-985707 | Fbxw32* | 0 |  |  |  |  |
| XLv80.chr2S:94679015-94679926 | Fbxl3 | 0 |  |  |  |  |
| XLv80.chr3L:7220946-7221590 | Fbxl3 | 2E-110 |  |  |  |  |
| XLv80.chr7S:71797637-71798130 | Fbxl4 | 0 |  |  |  |  |
| XLv80.chr4L:56833887-56834384 | Fbxl4 | 1E-108 |  |  |  |  |
| XLv80.chr1S:18346479-18347164 | Fbxl5 | 0 |  |  |  |  |
| XLv80.Sc004278_chrNA:1-436 | Fbxl7 | 0 |  |  |  |  |
| XLv80.chr1S:141493902-141494813 | Fbxl10 | 0 |  |  |  |  |
| XLv80.chr7S:42060525-42061524 | Fbxl11 | 0 |  |  |  |  |
| XLv80.chr3S:67679454-67679688 | Fbxl13 | 3E-63 |  |  |  |  |
| XLv80.Sc000054_chr8L:627351-628669 | Fbxl14 | 0 |  |  |  |  |
| XLv80.Sc000276_chrNA:13-1148 | Fbxl16 | 0 |  |  |  |  |
| XLv80.Sc000703_chrNA:16153-16691 | Fbxl16 | 5E-119 |  |  |  |  |
| XLv80.chr1S:152896923-152897400 | Fbxl17 | 2E-149 |  |  |  |  |
| XLv80.chr9_10S:2112362-2113128 | Fbxl20 | 4E-157 |  |  |  |  |
| XLv80.chr2S:94679017-94679661 | Fbxl21 | 3E-124 |  |  |  |  |
| XLv80.chr2L:110558189-110558833 | Fbxl21 | 1E-122 |  |  |  |  |
| XLv80.chr3L:75659055-75659521 | Fbxl22 | 2E-159 |  |  |  |  |
| XLv80.chr8L:71405346-71406055 | Fbxl23 | 0 |  |  |  |  |
| XLv80.chr9_10S:94552233-94552448 | Fbxo1 |  |  |  |  |  |
| XLv80.chr4L:10702282-10702758 | Fbxo3 | 1E-124 |  |  |  |  |
| XLv80.chr3S:2165896-2166378 | Fbxo7 | 1E-105 |  |  |  |  |
| XLv80.chr1S:36601123-36601698 | Fbxo8 | 0 |  |  |  |  |
| XLv80.Sc004423_chrNA:5583-5925 | Fbxo8 | 3E-143 |  |  |  |  |
| XLv80.chr5S:80000679-80001173 | Fbxo9 | 2E-102 |  |  |  |  |
| XLv80.chr1L:98335169-98335989 | Fbxo10 | 0 |  |  |  |  |
| XLv80.chr6L:68273813-68274110 | Fbxo15 | 1E-110 |  |  |  |  |
| XLv80.chr8L:59873209-59873600 | Fbxo17/27 | 7E-108 |  |  |  |  |
| XLv80.chr5L:9438311-9438496 | Fbxo28 | 8E-74 |  |  |  |  |
| XLv80.chr5S:43049458-43051272 | Fbxo30 | 0 |  |  |  |  |
| XLv80.chr6S:109839887-109840847 | Fbxo32 | 0 |  |  |  |  |
| XLv80.chr6L:111652248-111652900 | Fbxo32 | 0 |  |  |  |  |
| XLv80.chr8S:17445403-17446089 | Fbxo33 | 0 |  |  |  |  |
| XLv80.chr8S:6044707-6046287 | Fbxo34 | 0 |  |  |  |  |
| XLv80.Sc013782_chrNA:1141-1455 | Fbxo36 | 6E-115 |  |  |  |  |
| XLv80.chr2S:27802200-27802823 | Fbxo40 | 1E-130 |  |  |  |  |
| XLv80.chr1S:2697828-2698806 | Fbxo41 | 0 |  |  |  |  |
| XLv80.Sc001198_chrNA:1-262 | Fbxo42 | 1E-130 |  |  |  |  |
| XLv80.chr6S:98002740-98004187 | Fbxo43 | 0 |  |  |  |  |
| XLv80.chr8S:76229822-76231340 | Fbxo46 |  |  |  |  |  |
| No match | Fbxo47 |  |  |  |  |  |
| XLv80.chr2S:80450457-80450552 | Fbxo49* | 6E-33 |  |  |  |  |
| XLv80.chr7L:25508037-25508806 | Fbxw1 | 0 |  |  |  |  |
| XLv80.chr8S:30444888-30445401 | Fbxw2 | 0 |  |  |  |  |
| XLv80.chr1S:43656211-43656714 | Fbxw7 |  |  |  |  |  |
| XLv80.chr9_10L:42201409-42202329 | Fbxw10 | 0 |  |  |  |  |
| XLv80.chr9_10S:40304434-40305265 | Fbxw10 | 0 |  |  |  |  |
| XLv80.chr4S:6043271-6043995 | Fbxw29* | 3E-168 |  |  |  |  |
| XLv80.chr4L:984819-985509 | Fbxw29 | 3E-149 |  |  |  |  |
| XLv80.chr4L:984819-985707 | Fbxw30* |  |  |  |  |  |
| XLv80.chr4L:989998-991345 | Fbxw31* | 0 |  |  |  |  |
| XLv80.chr4S:6042786-6043964 | Fbxw32* | 0 |  |  |  |  |
| XLv80.chr4S:6019811-6020775 | Fbxw33* | 0 |  |  |  |  |
| XLv80.chr4L:984819-985707 | Fbxw33* | 0 |  |  |  |  |

|  | **Legend for color coding** |
| --- | --- |
|  | Expression from Xenbase |
|  | Expression identified in our analysis |
|  | Expression overlap with Xenbase and our analysis |
|  | Expression not detected by EST analysis and/or RT-PCR |
|  | Expression unknown |
